# Systems-Wide Dissection of Organic Acid Assimilation in Pseudomonas aeruginosa Reveals a Novel Path To Underground Metabolism

**DOI:** 10.1128/mbio.02541-22

**Published:** 2022-11-15

**Authors:** Stephen K. Dolan, Andre Wijaya, Michael Kohlstedt, Lars Gläser, Paul Brear, Rafael Silva-Rocha, Christoph Wittmann, Martin Welch

**Affiliations:** a School of Biological Sciences, Georgia Institute of Technology, Atlanta, Georgia, USA; b Emory–Children’s Cystic Fibrosis Center, Atlanta, Georgia, USA; c Center for Microbial Dynamics and Infection, Georgia Institute of Technology, Atlanta, Georgia, USA; d Centre for Targeted Protein Degradation, University of Dundee, Dundee, Scotland, United Kingdom; e Institute of Systems Biotechnology, Saarland University, Saarbrücken, Germany; f Department of Biochemistry, University of Cambridge, Cambridge, United Kingdom; g Faculdade de Medicina de Ribeirão Preto, Universidade de São Paulo, São Paulo, Brazil; Geisel School of Medicine at Dartmouth

**Keywords:** *Pseudomonas aeruginosa*, enzyme promiscuity, 2-methylcitrate cycle, central metabolism, propionate metabolism, underground metabolism

## Abstract

The human pathogen Pseudomonas aeruginosa (Pa) is one of the most frequent and severe causes of nosocomial infection. This organism is also a major cause of airway infections in people with cystic fibrosis (CF). Pa is known to have a remarkable metabolic plasticity, allowing it to thrive under diverse environmental conditions and ecological niches; yet, little is known about the central metabolic pathways that sustain its growth during infection or precisely how these pathways operate. In this work, we used a combination of ‘omics approaches (transcriptomics, proteomics, metabolomics, and ^13^C-fluxomics) and reverse genetics to provide systems-level insight into how the infection-relevant organic acids succinate and propionate are metabolized by Pa. Moreover, through structural and kinetic analysis of the 2-methylcitrate synthase (2-MCS; PrpC) and its paralogue citrate (CIT) synthase (GltA), we show how these two crucial enzymatic steps are interconnected in Pa organic acid assimilation. We found that Pa can rapidly adapt to the loss of GltA function by acquiring mutations in a transcriptional repressor, which then derepresses *prpC* expression. Our findings provide a clear example of how “underground metabolism,” facilitated by enzyme substrate promiscuity, “rewires” Pa metabolism, allowing it to overcome the loss of a crucial enzyme. This pathogen-specific knowledge is critical for the advancement of a model-driven framework to target bacterial central metabolism.

## INTRODUCTION

Pseudomonas aeruginosa (Pa) is a notorious opportunistic human pathogen that frequently infects the airways of people with cystic fibrosis (pwCF). Pa is also well known for being metabolically flexible. This flexibility is important because nutrient acquisition and assimilation during infection scenarios are likely to be complex and dynamic processes. Indeed, there is an increasing realization that metabolic enzymes may also serve as targets for the next generation of antimicrobial therapies (1). However, we currently lack a clear understanding of how core metabolism operates in Pa.

Laboratory strains of Pa are known to prefer C-4-dicarboxylates, such as malate (MAL), fumarate, and succinate, as carbon and energy sources during growth *in vitro* (2). However, during infection scenarios, Pa frequently has to use less-favored carbon sources for growth, such as the host-derived airway surfactant phosphatidylcholine (PC). This phospholipid is broken down by secreted Pa phospholipases to yield phosphorylcholine, glycerol, and long-chain fatty acids (3, 4).

**FIG 1:**
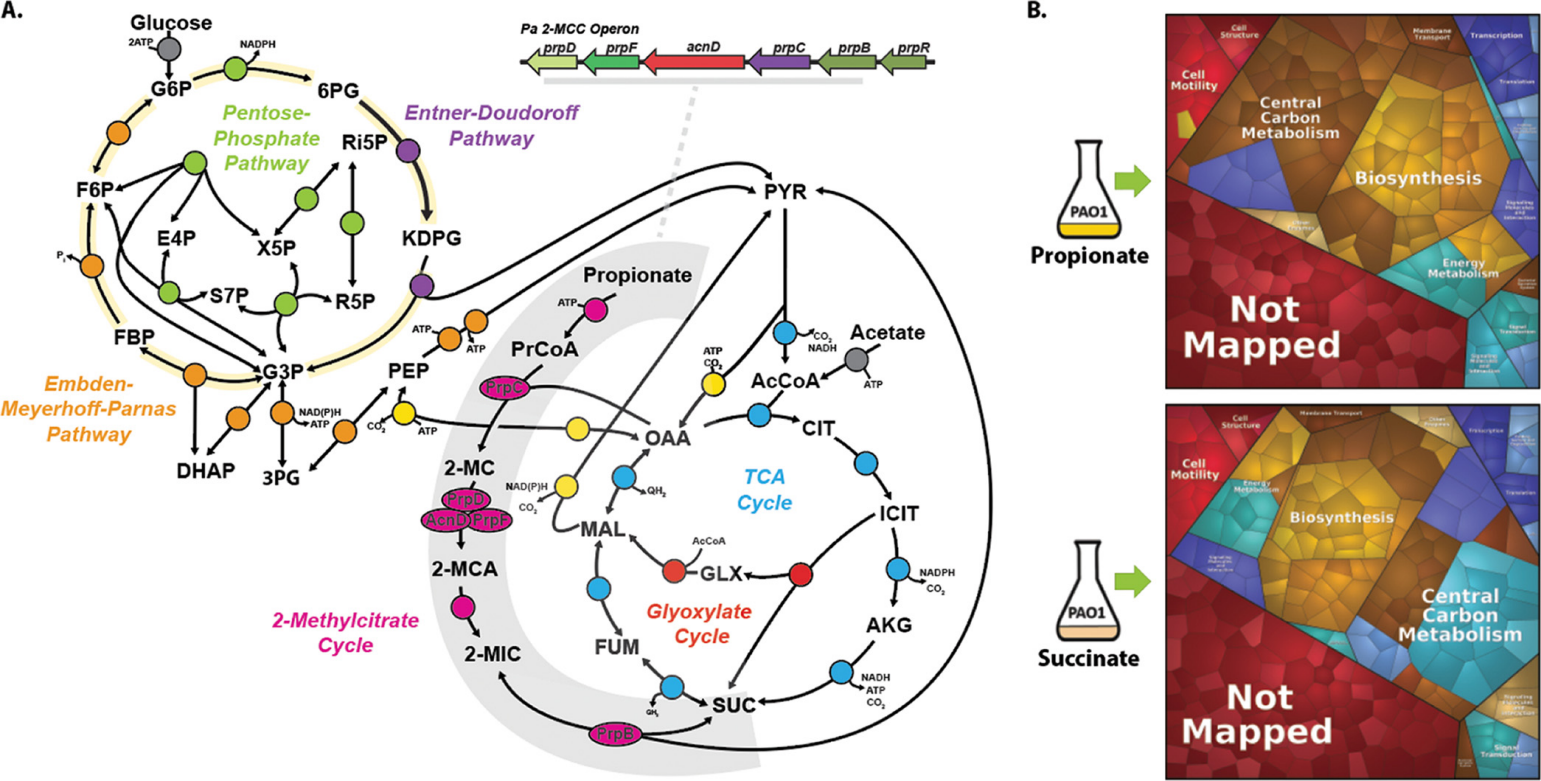
Proteomic analysis of Pa grown on succinate and propionate. (A) Schematic depicting the Pa 2-methylcitrate cycle (2MCC) in Pa central carbon metabolism. The Pa central metabolic network shown here consists of six main blocks, designated with different colors: (i) the Embden-Meyerhoff-Parnas pathway (EMP; orange); (ii) the pentose phosphate pathway (PPP; green); (iii) the Entner-Doudoroff pathway (EDP; purple); (iv) the tricarboxylic acid cycle (TCA; blue) and glyoxylate shunt (red); (v) anaplerotic and gluconeogenic reactions (yellow); and (vi) the 2MCC (pink). The 2MCC operon arrangement (inset, gray underline) consists of genes that encode a transcriptional regulator (designated here as *prpR*), which is thought to encode a ligand-responsive repressor, a methylcitrate synthase (*prpC*), which condenses propionyl-CoA (PrCoA) with oxaloacetate (OAA) to form 2-methylcitrate (2-MC), a 2-methylcitrate dehydratase/hydratase (*prpD*), which dehydrates 2-MC to yield 2-methylaconitate (2-MCA), a 2-methylcitrate dehydratase (*acnD*) and 2-methylaconitate *cis-trans* isomerase (*prpF*), which provide an alternative route for the generation of 2-MCA from 2-MC (the reason for an alternative route for 2-MCA generation in Pa is currently unclear), and a 2-methylisocitrate lyase (*prpB*), which cleaves 2-methylisocitrate (2-MIC) to yield pyruvate (PYR) and succinate (SUC). Note that the 2-MCA generated in the PrpD or AcnD/PrpF reactions is rehydrated by an unlinked aconitase (likely AcnB in Pa) to yield the PrpB substrate 2-MIC. Also, the enzyme responsible for the initial activation of propionate to yield PrCoA has not yet been identified for Pa, although in other organisms this function is carried out by a dedicated propionyl-CoA synthase (PrpE), by acetyl-CoA synthase (AcsA), by a combination of phosphotransacetylase (Pta) and acetate kinase (AckA) activities, or by an additional, uncharacterized propionyl-CoA ligase (7). AcCoA, acetyl-coenzyme A; CIT, citrate; ICIT, isocitrate; AKG, α-ketoglutarate; FUM, fumarate; MAL, malate; KDPG, 2-keto-3-deoxy-6-phosphogluconate; G3P, glyceraldehyde 3-phosphate; FBP, fructose 1,6-bisphosphate; F6P, fructose 6-phosphate; G6P, glucose 6-phosphate; 6PG, 6-phosphogluconate; Ri5P, ribulose 5-phosphate; R5P, ribose 5-phosphate; X5P, xylulose 5-phosphate; S7P, sedoheptulose 7-phosphate; E4P, erythrose 4-phosphate; PEP, phosphoenolpyruvate. (B) Illustration of the statistically significant proteomic changes (*P* ≤ 0.05, fold change of ≥1) during growth on propionate or succinate, as represented by Voronoi tessellations. Pathway assignment was performed using the KEGG data set. Proteome alterations that could not be assigned to a specific pathway (uncharacterised/hypothetical proteins) are shown as “Not Mapped.” The specific protein identities for the protein clusters that were upregulated during growth on propionate are shown in Fig. S1A in the supplemental material, and statistical analyses of these data are illustrated in Fig. S1B to D. The complete proteomics data set is presented in Data Set S1.

Pa can also metabolize short-chain fatty acids. Propionate is a naturally occurring short-chain fatty acid produced by the human gut microbiota and is a commonly used food preservative with potent bacteriostatic activity. Another rich source of propionate are the anaerobes that frequently occupy the lower airways of pwCF. These anaerobes break down tracheobronchial mucin to produce copious quantities of propionate. However, and in spite of its known growth-inhibitory properties against some species of bacteria, Pa is able to thrive on propionate and can very effectively utilize the compound as a sole carbon source *in vitro* (5–7). Pa does this by catabolizing propionate through the 2-methylcitrate (2-MC) cycle (2MCC) (Fig. 1A) to yield succinate and pyruvate (PYR), which feed directly into the tricarboxylic acid (TCA) cycle. The 2MCC also sits at an important junction in amino acid catabolism, as several amino acids (l-valine, *l-isoleucine*, l-methionine, and l-threonine) are degraded to propionyl-coenzyme A (propionyl-CoA [PrCoA]), which must then be oxidized by this pathway (7). Given the ubiquity of propionate in many host niches, it comes as little surprise that a functional 2MCC is required for infection by a plethora of human pathogens, including Mycobacterium tuberculosis, Neisseria meningitides, Aspergillus fumigatus, and Talaromyces marneffei (8–12). The Pa 2MCC has also been shown to be important for infection of the nematode intestine (13).

10.1128/mbio.02541-22.1 FIG S1 Proteomic analysis of Pa cultured in MOPS-succinate versus MOPS-propionate. (A) A Voronoi tessellation illustrating the proteins that are increased in abundance during exponential growth of PAO1 in MOPS-propionate compared with growth in MOPS-succinate. Notable proteins include those in the ORF cluster (PA3232 to PA3235, blue) containing the putative ActP transporter protein (PA3234), enzymes in the 2MCC, glyoxylate shunt and TCA cycle (brown), cytochrome oxidase components (turquoise), and enzymes involved in branched-chain amino acid catabolism (tan). (B) Volcano plot illustrating log_2_ fold change in Pa protein abundance versus adjusted *P* values for the MOPS-propionate versus MOPS-succinate data sets. (C) Principal component analysis (PCA) of the proteomic data from the Pa grown in MOPS-propionate (red) and MOPS-succinate (blue). (D) Box and whisker plot illustrating the normalized MOPS-propionate (yellow) and MOPS-succinate (blue) replicates. (E) Pa pyruvate carboxylase (*pycA*::*Tn* and *pycB*::*Tn*) transposon mutants cultured on MOPS-succinate agar or MOPS-propionate agar (as indicated) alongside PAO1 and Δ*prpC*. Note that the pyruvate carboxylase mutants cannot grow on propionate as a sole carbon source. The plates were photographed after 24 h of incubation. The data are representative of two independent experiments performed in triplicate. (F to I) Luciferase activity in P. aeruginosa PAO1 carrying chromosomal promoter::*lux* fusions for the indicated promoters cultured in MOPS-succinate, MOPS-propionate, MOPS-glucose, or MOPS-acetate: *acsA* (F), *cco2* (G), *aceA* (H), and *glcB* (I). Values are normalized to OD_600_ (RLU/OD_600_). The data represent three biological replicates per sample. The data were analyzed using GraphPad Prism (V 6.01), and statistical significance was determined with an unpaired parametric *t* test with Welch’s correction. Statistically significant differences in RLU/OD_600_ are indicated; ns, *P* > 0.05; *, *P* ≤ 0.05; **, *P* ≤ 0.01; ***, *P* ≤ 0.001. Download FIG S1, PNG file, 1.7 MB. Copyright © 2022 Dolan et al. 2022 Dolan et al. https://creativecommons.org/licenses/by/4.0/ This content is distributed under the terms of the Creative Commons Attribution 4.0 International license.

10.1128/mbio.02541-22.8 DATA SET S1 Summary of electron transport chain- and central metabolism-associated ‘omic changes described in the main text (tab A) and full proteomics (tab B) of MOPS-propionate versus MOPS-succinate grown Pa. Download Data Set S1, XLSX file, 0.3 MB. Copyright © 2022 Dolan et al. 2022 Dolan et al. https://creativecommons.org/licenses/by/4.0/ This content is distributed under the terms of the Creative Commons Attribution 4.0 International license.

We currently have a limited understanding of how Pa metabolizes propionate or how the 2MCC interfaces with the other components of central carbon metabolism in this organism. Although some features of the pathway can be extrapolated from a knowledge of the biochemistry in other bacteria (such as Escherichia coli and Salmonella enterica), these are fundamentally dissimilar microbes with alternative operonic arrangements for the 2MCC open reading frames (ORFs) and very different metabolic architectures compared with Pa (14, 15). For example, propionate metabolism in several *Enterobacteriales* (including E. coli and S. enterica) and all analyzed *Xanthomonadales* is coordinated by a Fis family transcription factor (TF) known as PrpR (16). By contrast, the 2MCC in *Gammaproteobacteria* is typically controlled by a GntR family TF. Remarkably, no 2MCC regulators from the GntR family have been experimentally characterized to date. Therefore, and to understand better how Pa utilizes propionate, we used a combination of ‘omics approaches (transcriptomics, proteomics, metabolomics, and ^13^C-fluxomics) and reverse genetics to provide a systems-level insight into how the organic acids succinate and propionate are metabolized by Pa. Moreover, through structural and kinetic analysis of the 2-methylcitrate synthase (PrpC) and its paralogue citrate synthase (GltA), we show how these two crucial steps are interconnected in organic acid assimilation. Building on these observations, we found that Pa can rapidly adapt to the loss of GltA by acquiring mutations that derepress expression of the *prpC*-encoding 2MCC operon (*prp*). These mutations are in a GntR-family TF, which we show encodes a transcriptional repressor of the *prp* operon. Our findings provide a clear example of how “underground metabolism” (17), facilitated by enzyme promiscuity, allows Pa to overcome the loss of a crucial enzyme in central carbon metabolism.

## RESULTS

### ‘Omics-driven examination of Pa grown on succinate and propionate as sole carbon sources.

To understand how growth on different substrates affects the physiology of Pa, we first examined the proteome during exponential-phase growth on succinate or on propionate as a sole carbon source. Through proteomic analysis, we identified and quantified 3,796 proteins. Of these, 265 proteins showed increased abundance during growth on propionate, and 295 proteins showed increased abundance during growth on succinate (*q* ≤ 0.05, log_2_ fold change (FC) ≥ 1 or ≤ −1; Data Set S1 in the supplemental material). To obtain a global overview of the physiological changes, we used the Proteomaps web service (18) to generate Voronoi tessellations (19) structured around the KEGG orthologies of the statistically significant changes (*P* ≤ 0.01, log_2_ fold change ≥1 or ≤ −1). As shown in Fig. 1B, most of the proteomic changes were associated with “central carbon metabolism,” “biosynthesis,” “signaling and cellular process,” and “energy metabolism.” Notably, growth on propionate led to a strong induction (~16-fold change) of all proteins encoded by the *prp* operon, including the GntR-family 2MCC operon regulator PA0797, which we designate here as PrpR (Fig. S1A to D; Data Set S1A).

**FIG 2:**
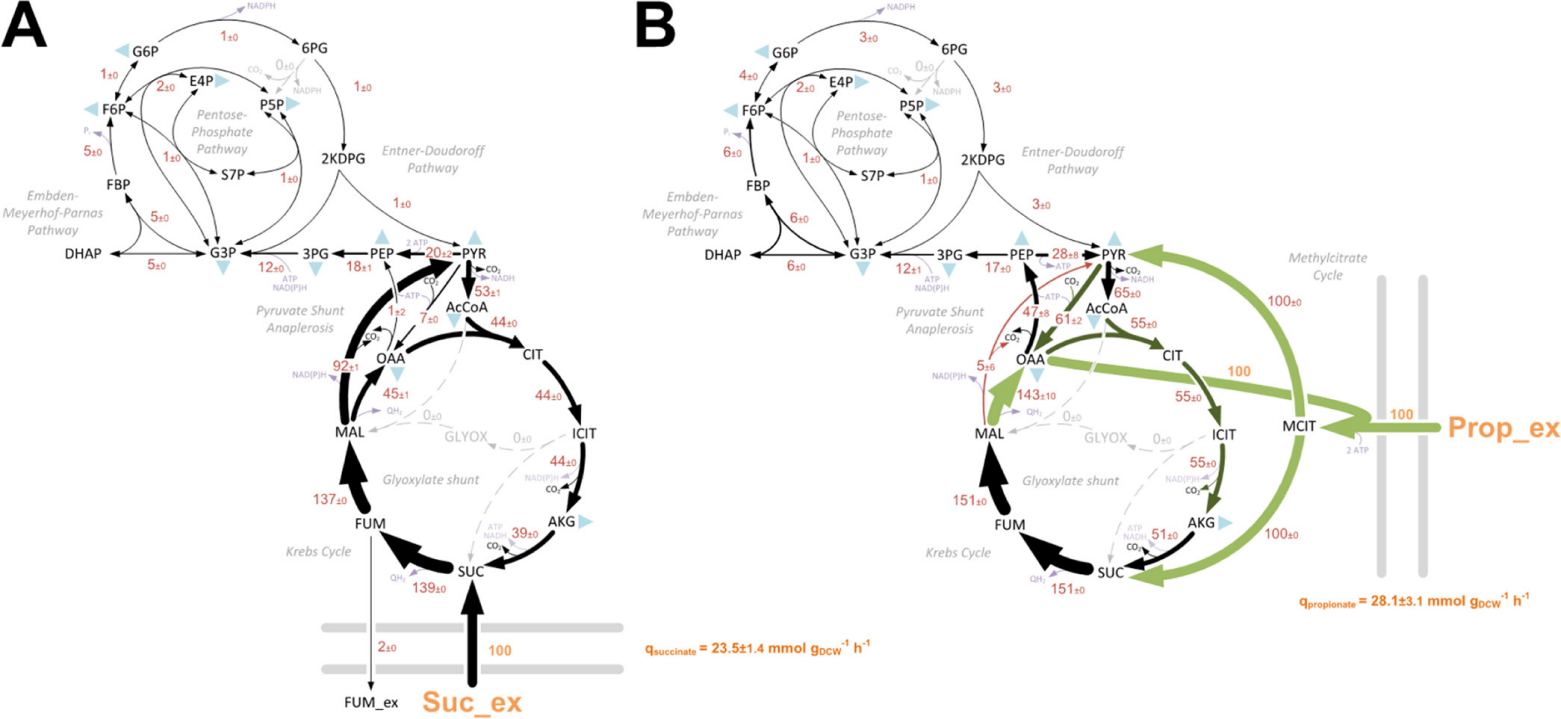
(A and B) *In vivo* carbon flux distributions in central metabolism of Pa PAO1 during growth on succinate (A) or propionate (B) as sole carbon sources. Flux is expressed as a molar percentage of the average uptake rate for succinate (23.5 mmol g^−1^ h^−1^) or propionate (28.1 mmol g^−1^ h^−1^), calculated from the individual rates in Data Set S2 in the supplemental material. Anabolic pathways from 11 precursors to biomass are indicated by the filled blue triangles. The flux distributions with bidirectional resolution (i.e., net and exchange fluxes), including the drain from metabolic intermediates to biomass and confidence intervals of the flux estimates, are provided in Data Set S2. The errors given for each flux reflect the corresponding 90% confidence intervals. The full flux data sets are presented in Data Set S2. Colors qualitatively indicate fluxomic correlation with changes on the protein level during growth on propionate compared with growth on succinate (light green or red, significant up- or downregulation (respectively); dark green or red, less significant up- or downregulation).

To provide a complementary insight into the absolute metabolic fluxes in Pa during growth on propionate and succinate, we also carried out a [^13^C] fluxome analysis. This was achieved by measuring the mass isotopomer distributions in proteinogenic amino acids and cell carbohydrates using three separate tracers for propionate and succinate (Materials and Methods) (20). The calculated relative fluxes for Pa strain PAO1 grown on labeled propionate or succinate are shown in Fig. 2. The corresponding quantitative comparison of NADPH (redox) supply and ATP (energy) supply for succinate- and propionate-grown Pa are shown in Fig. S2.

10.1128/mbio.02541-22.2 FIG S2 Quantitative analysis of redox and energy supply and demand for Pa grown on succinate (A and C) or propionate (B and D) as a sole carbon source. Reactions linked to NADPH (A and B) and ATP (C and D) metabolism were calculated from the obtained fluxes (Fig. 2). Values are given as absolute fluxes (mmol g^−1^ h^−1^) and are related to the specific carbon uptake rate (Data Set S2). G6PDH, glucose 6-phosphate dehydrogenase; MAE, malic enzyme; ICDH, isocitrate dehydrogenase(s); G3PDH, glyceraldehyde 3-phosphate dehydrogenase; SCS, succinyl-CoA synthase; Ox-P, oxidative phosphorylation. Download FIG S2, PNG file, 0.5 MB. Copyright © 2022 Dolan et al. 2022 Dolan et al. https://creativecommons.org/licenses/by/4.0/ This content is distributed under the terms of the Creative Commons Attribution 4.0 International license.

10.1128/mbio.02541-22.9 DATA SET S2 [^13^C] fluxomics data for Pa grown in MOPS-propionate or MOPS-succinate, including OpenFLUX SimVector files (tab A), calculations for anabolic demand (tab B), reaction network (tab C), goodness of fit (tab D), and metabolic fluxes (tab E), CoA detection (tab F), and organic acid uptake (tab G). Download Data Set S2, XLSX file, 0.1 MB. Copyright © 2022 Dolan et al. 2022 Dolan et al. https://creativecommons.org/licenses/by/4.0/ This content is distributed under the terms of the Creative Commons Attribution 4.0 International license.

Comparison of the flux maps, in combination with the proteomic data, generated an unparalleled insight into the central carbon metabolic networks of Pa during growth on both substrates. For example, several of the key proteomic alterations found when comparing growth on propionate with growth on succinate were in core central carbon metabolism (Data Set S1A). These core changes were largely consistent with the corresponding carbon flux distributions (Fig. 2). In general, the expression of enzymes from the pentose phosphate pathway (PPP), the Embden-Meyerhof-Parnas pathway (EMPP), and the Entner-Doudoroff pathway (EDP) was decreased during growth in propionate compared with during growth in succinate. The expression of several enzymes in the TCA cycle was increased during growth on propionate, including citrate synthase GltA (2.5 FC), aconitase AcnA (2.3 FC), and the isocitrate dehydrogenases ICD (2.5 FC) and IDH (1.7 FC). A corresponding increase in TCA cycle carbon flux was also evident, with roughly an 11% increase in flux through the reactions between citrate (CIT) and malate (MAL) (Fig. 2). Fumarate efflux (2%) was also detected during Pa growth using succinate as a sole carbon source.

Among the largest discrepancies between the propionate- and succinate-grown cultures at both the proteome and fluxome levels were noted at the reactions involved in the pyruvate shunt. Expression of the malic enzyme MaeB, which catalyzes the oxidative decarboxylation of malate to produce pyruvate and CO_2_, was downregulated (−4.7 FC) during growth on propionate. By contrast, the pyruvate carboxylase-encoding genes *pycA* (PA5435) and *pycB* (PA5436), which catalyze the ATP-dependent carboxylation of pyruvate to yield oxaloacetate (OAA), were upregulated (2.6 FC) as was the regulator PycR (21, 22). These alterations matched the corresponding flux data, which revealed a substantial decrease in flux from malate to pyruvate (−87%) and an increase in the flux from pyruvate to OAA (54%) during growth on propionate. There is a good metabolic logic to this. Although the catabolism of propionate yields succinate and pyruvate, an early enzyme in the propionate catabolic pathway (PrpC) requires oxaloacetate as a substrate (Fig. 1). Therefore, we hypothesized that this drain on the oxaloacetate pool may be countered by a combination of lower malic enzyme-mediated pyruvate generation and increased anaplerotic pyruvate carboxylase activity to sustain the TCA cycle. In support of this, mutants defective in pyruvate carboxylase (encoded by *pycA* and *pycB*) were unable to grow on propionate as a sole carbon source (Fig. S1E). Growth on propionate also increased the expression (5.1 FC) of the membrane-bound malate-quinone oxidoreductase MqoB, which generates oxaloacetate directly from malate. A corresponding 98% increase in carbon flux from malate to oxaloacetate was evident during growth on propionate (Fig. 2).

The expression level of phosphoenolpyruvate (PEP) synthase (PPS) and pyruvate kinase showed no significant differences between the growth conditions. However, the fluxomic analysis captured a pronounced alteration in carbon flow at this node. During growth on succinate, the net carbon flux was in the pyruvate → phosphoenolpyruvate (gluconeogenic) direction, with phosphoenolpyruvate originating from pyruvate mainly via the combined activity of malic enzyme (Mae) and PEP synthase at the equivalent cost of 2 ATP (PYR + H_2_O + ATP → PEP + AMP + P_i_). By contrast, during growth on propionate, the net flux at this node was in the direction phosphoenolpyruvate → pyruvate, a reaction that is catalyzed by pyruvate kinase isozyme A (PykA) and generates ATP (23). In this scenario, phosphoenolpyruvate largely originates from the action of phosphoenolpyruvate carboxykinase (PckA) on the oxaloacetate that is generated via the activity of malate-quinone oxidoreductase (MqoB). Interestingly, and despite the greatly increased flux from oxaloacetate to phosphoenolpyruvate catalyzed by PckA, the expression of this enzyme was decreased 1.7-fold during growth on propionate compared with growth on succinate. This may indicate a role for allosteric regulation in modulating PckA enzyme activity (24, 25).

Compared with growth on succinate, the glyoxylate shunt enzymes isocitrate lyase (ICL; AceA; 5.5 FC) and malate synthase (GlcB; 3.6 FC) were highly expressed on propionate. This was also verified using promoter-luciferase transcriptional fusions (Fig. S1F to I). However, the fluxomics data indicated that there was no carbon flux through the glyoxylate shunt during growth on either succinate or propionate as a sole carbon source. This may be explained by the extensive allosteric interactions that are known to control flux partitioning between the TCA cycle and glyoxylate shunt. ICL activity in Pa is allosterically inhibited by oxaloacetate, pyruvate, succinate, phosphoenolpyruvate (PEP), and CoA. By contrast, oxaloacetate and pyruvate allosterically activate one of the isocitrate dehydrogenase enzymes IDH (26). As flux to pyruvate is significantly increased during growth on either succinate or propionate compared with acetate (where flux through the glyoxylate shunt is maximal), these data suggest that pyruvate is the most likely metabolite responsible for abrogating flux through the glyoxylate shunt during growth on propionate (27).

Several studies have suggested that diffusion across the cytoplasmic membrane is a major mechanism of both acetate and propionate uptake in bacteria (28, 29). Dedicated transport mechanisms for monocarboxylic acids have also been described (30). One cluster of ORFs (PA3232 to PA3235) was upregulated (~16 FC) during growth on propionate and encode a putative acetate permease (ActP). Indeed, PA3234 shows 80% amino acid identity to the ActP protein from Escherichia coli, and this ORF has been previously shown to be regulated by the two-component system MxtR*/*ErdR, which is essential for growth on acetate (31, 32). MxtR was also upregulated during growth on propionate (8.7 FC), whereas proteins associated with dicarboxylic acid transport (DctA, DctQ, DctP, and PA5530) were more abundant during growth on succinate (Data Set S1A) (33, 34).

Aerobic growth in different carbon sources results in large-scale remodeling of the electron transport chain in Pa, including components of the denitrification pathway (27). Growth on propionate led to significantly increased expression of most terminal oxidases, particularly the quinol oxidase Cyo (7.6 FC), the cyanide-insensitive oxidase Cio (5.4 FC), the cytochrome c oxidase Cco2 (3.0 FC), and the cytochrome oxidase Cox (2.2 FC). Furthering the notion of an altered redox balance during growth on the two substrates, we noticed differences in expression of the NAD(P) transhydrogenases, which fine-tune the size and degree of reduction of the NAD pools (35). Expression of the transhydrogenase Sth (PA2991), which is thought to primarily convert NADPH to NADH, was increased during growth on propionate (2.1 FC), whereas the transhydrogenase proteins PntAA (3.6 FC) and PntB (2.2 FC) (which presumably catalyze the interconversion of NADH to NADPH) were more abundant during growth on succinate. These alterations were reflected in the redox balances; growth on propionate resulted in a lower NADPH surplus (as any excess is assumed to be converted to NADH to drive ATP synthesis) than growth on succinate (Fig. S2).

The 2MCC also serves a role in the catabolism of branched-chain amino acids (BCAA). This is because isoleucine and valine degradation generates propionyl-CoA, which can then be degraded to succinate and pyruvate via the 2MCC (36). The metabolism of valine produces the intermediate (*S*)-3-hydroxyisobutyric acid, which is oxidized to methylmalonate semialdehyde by 3-hydroxyisobutyrate dehydrogenase (MmsB). Methylmalonate semialdehyde dehydrogenase (MmsA) then catalyzes the irreversible NAD^+^- and CoA-dependent oxidative decarboxylation of the semialdehyde to yield propionyl-CoA (36, 37). Expression of MmsB (28.1 FC) and MmsA (5.3 FC) was significantly increased during growth on propionate. This suggests that there is a regulatory link between the 2MCC and BCAA catabolism in Pa.

### Propionate inhibits the growth of Pa when propionate catabolism is disrupted.

**FIG 3:**
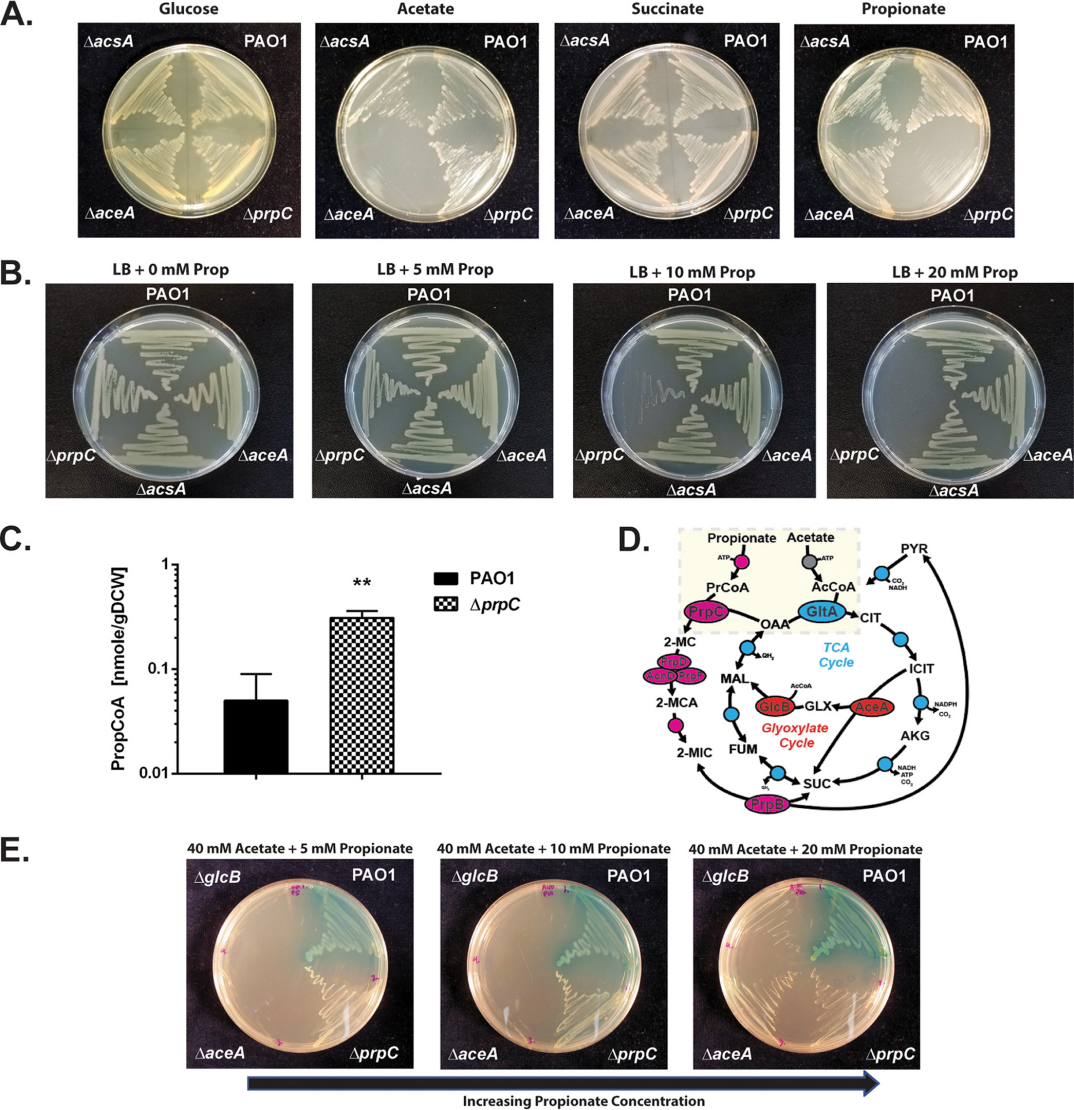
The Pa ORF (*prpC*) encoding 2-methylcitrate synthase is essential for growth on propionate. (A) Wild-type Pa (PAO1) and the Δ*acsA*, Δ*aceA*, and Δ*prpC* mutants all grow comparably on MOPS agar containing glucose (20 mM) or succinate (30 mM) as a sole carbon source. The Δ*acsA* mutant has a growth defect during growth on MOPS-acetate (40 mM) and MOPS-propionate (40 mM). The Δ*prpC* mutant cannot grow on MOPS propionate. The plates were photographed after 24 h of incubation. (B) Wild-type PAO1 and the Δ*acsA*, Δ*aceA*, and Δ*prpC* mutants were cultured on LB agar containing an increasing concentration of propionate (0, 5, 10, and 20 mM, as indicated). The Δ*prpC* mutant displays a pronounced growth defect in the presence of propionate concentrations of >10 mM. The plates were photographed after 24 h of incubation. (C) Intracellular propionyl-CoA concentration in wild-type Pa (PAO1) and in the Δ*prpC* mutant following a 3-h exposure to propionate (5 mM) during growth in succinate (unpaired *t* test with Welch’s correction, *P* = 0.0026). The experiment was performed using biological triplicates. (D) Illustration of the interwoven reactions for propionate and acetate activation in Pa, feeding into the 2-methylcitrate cycle and TCA cycle, respectively. Following uptake, acetate and propionate are activated by AcsA. The resulting propionyl-CoA (PrCoA) is condensed with oxaloacetate (OAA) in a PrpC-catalyzed reaction to form 2-methylcitrate (2-MC), whereas the acetyl-CoA (AcCoA) is condensed with oxaloacetate in a GltA-catalyzed reaction to form citrate (CIT). (E) Growth of the Pa glyoxylate shunt mutants Δ*aceA* and Δ*glcB* is blocked on MOPS agar plates containing a combination of high acetate concentration (40 mM) and low propionate concentration (5 mM) as the carbon source. However, this growth inhibition is partially overcome by increasing the propionate concentration to 20 mM (left to right in the figure). The plates were photographed after 48 h of growth. The data are representative of two independent experiments, each performed in triplicate.

Based on the proteomics data, we made (separate) in-frame deletions in a selection of genes putatively involved in propionate uptake (PA3234, *actP* homologue), propionate activation (*acsA*, PA3568), and propionate catabolism (*prpC*, *mmsA*, *aceA*, and *glcB*) and tested the ability of the resulting mutants to grow on a series of single carbon sources (Fig. 3A; Fig. S3A to C). Importantly, the acetyl-CoA (AcCoA) synthetase mutant (Δ*acsA*) exhibited a pronounced growth defect with either acetate or propionate as a sole carbon source, suggesting that propionate may be a secondary substrate for this enzyme (Fig. S3B and C). By contrast, the ΔPA3568 mutant (defective in another potential propionyl-CoA synthetase) exhibited no phenotype during growth on propionate or acetate. This suggests that additional, currently uncharacterized propionyl-CoA ligase(s) may be present in Pa. Similarly, mutants defective in *mmsA*, *aceA*, or *glcB* or in the putative acetate symporter (ΔPA3234) also exhibited no growth defects on propionate, despite significant upregulation of the corresponding gene products during growth on this carbon source. The 2-methylcitrate synthase mutant (Δ*prpC*) was unable to grow on propionate as a sole carbon source but displayed no detectable growth deficit on any of the other tested carbon sources.

10.1128/mbio.02541-22.3 FIG S3 Growth characteristics of selected mutants in MOPS-succinate, MOPS-propionate, and MOPS-branched-chain amino acids media. (A) Growth curves of PAO1 and the Δ*prpC*, ΔPA3568, Δ*mmsB*, ΔPA3234, and Δ*acsA* mutants cultured in MOPS-succinate (30 mM). Data are representative of three independent experiments performed in triplicate. (B) Growth curves of PAO1 and the Δ*aceA*, Δ*glcB*, ΔPA3568, Δ*mmsB*, and Δ*acsA* mutants cultured in MOPS-propionate (40 mM). Data are representative of three independent experiments performed in triplicate. (C) Growth curves of PAO1 and the Δ*prpC*, ΔPA3234, ΔPA3568, Δ*mmsB*, and Δ*acsA* mutants cultured in MOPS-acetate (40 mM). Data are representative of three independent experiments performed in triplicate. (D) Growth curves of PAO1 and the Δ*prpC* and ΔPA3234 mutants cultured in MOPS-succinate (30 mM) containing 0 mM, 5 mM, and 10 mM propionate. Data are representative of three independent experiments performed in triplicate. (E) Growth of PAO1 and the Δ*prpC*, ΔPA3568, Δ*mmsA*, ΔPA3234, Δ*acsA*, and Δ*ackA* mutants cultured on MOPS + branched-chain amino acids (BCAA) as a sole carbon source (2 mM each of l-isoleucine, l-valine, and l-leucine). The plates were photographed after 48 h. Data are representative of two independent experiments performed in triplicate. (F) Growth of PAO1 and the Δ*aceA*, Δ*glcB*, and Δ*prpC* mutants cultured on 5 mM MOPS propionate, 40 mM acetate, and 40 mM MOPS-acetate + 5 mM succinate. The plates were photographed after 48 h. The data are representative of two independent experiments performed in triplicate. Download FIG S3, PNG file, 1.5 MB. Copyright © 2022 Dolan et al. 2022 Dolan et al. https://creativecommons.org/licenses/by/4.0/ This content is distributed under the terms of the Creative Commons Attribution 4.0 International license.

It has been established for several microorganisms that in the absence of a functional 2-methylcitrate synthase (PrpC), propionate (derived either through direct catabolism of propionate or through the catabolism of branched-chain amino acids) has growth-inhibitory properties (38–40). It is likely that this toxicity is mediated by downstream pathway intermediates, such as 2-MC isomers, which could be generated from the accumulated intracellular propionyl-CoA (39).

As shown in Fig. 3B, when propionate (5 to 20 mM) is added to lysogeny broth (LB) agar, growth of the Δ*prpC* mutant becomes progressively more inhibited as the concentration of propionate increases. The growth-inhibitory effect of propionate on the Δ*prpC* mutant was also apparent when propionate was added to morpholinepropanesulfonic acid (MOPS)-buffered succinate, ruling out pH-dependent toxicity (Fig. S3D). Notably, the Δ*prpC* mutant was also unable to grow on branched-chain amino acids as a sole carbon source (Fig. S3E). To examine the possible basis for growth inhibition following propionate exposure further, we exposed PAO1 and the Δ*prpC* mutant to 5 mM propionate during exponential growth on succinate (25 mM). Then, after a further 3 h of growth, we measured the intracellular propionyl-CoA levels in each sample (Fig. 3C). This revealed that even during growth on a preferred carbon source, succinate, propionyl-CoA accumulates in the Δ*prpC* mutant compared with wild-type PAO1.

We previously characterized the metabolic pathways expressed in Pa during growth on acetate (27). Comparison of those data with the results presented here for growth on propionate revealed several commonalities, including increased expression of AcsA, PA3234 (the *actP* homologue), and the glyoxylate shunt enzymes on acetate and propionate. This may reflect the activity of shared regulators or analogous reaction mechanisms and overlapping substrates (Fig. 3D). As shown in Fig. S3F and consistent with the fluxomics data (which revealed negligible flux through the glyoxylate shunt during growth on propionate), mutants defective in the glyoxylate shunt enzymes, Δ*aceA* and Δ*glcB*, suffered no growth defect on propionate as a sole carbon source. As expected, the same mutants were unable to grow on acetate as a sole carbon source (Fig. S3F). Remarkably, this growth of the Δ*aceA* and Δ*glcB* mutants on propionate was blocked when acetate was added to the medium (Fig. 3E). This growth inhibition could be partially relieved by increasing the concentration of propionate in the medium, suggesting metabolic competition between acetate and propionate catabolism (Fig. 3E). By contrast, acetate did not prevent growth of the Δ*aceA* and Δ*glcB* mutants on plates containing succinate (Fig. S3F). In the absence of the glyoxylate shunt, acetyl-CoA generated through the activation of acetate or through β-oxidation of fatty acids is unable to contribute to Pa biomass generation (41). Because AcsA likely activates both acetate and propionate, a parsimonious hypothesis is that saturating concentrations of acetate (which is probably the preferred substrate of AcsA) competitively block the activation of propionate. This competition is relieved at higher propionate concentrations, thereby enabling growth of the Δ*aceA* and Δ*glcB* mutants.

### Structural and functional investigation of PrpC and GltA from Pa.

**FIG 4:**
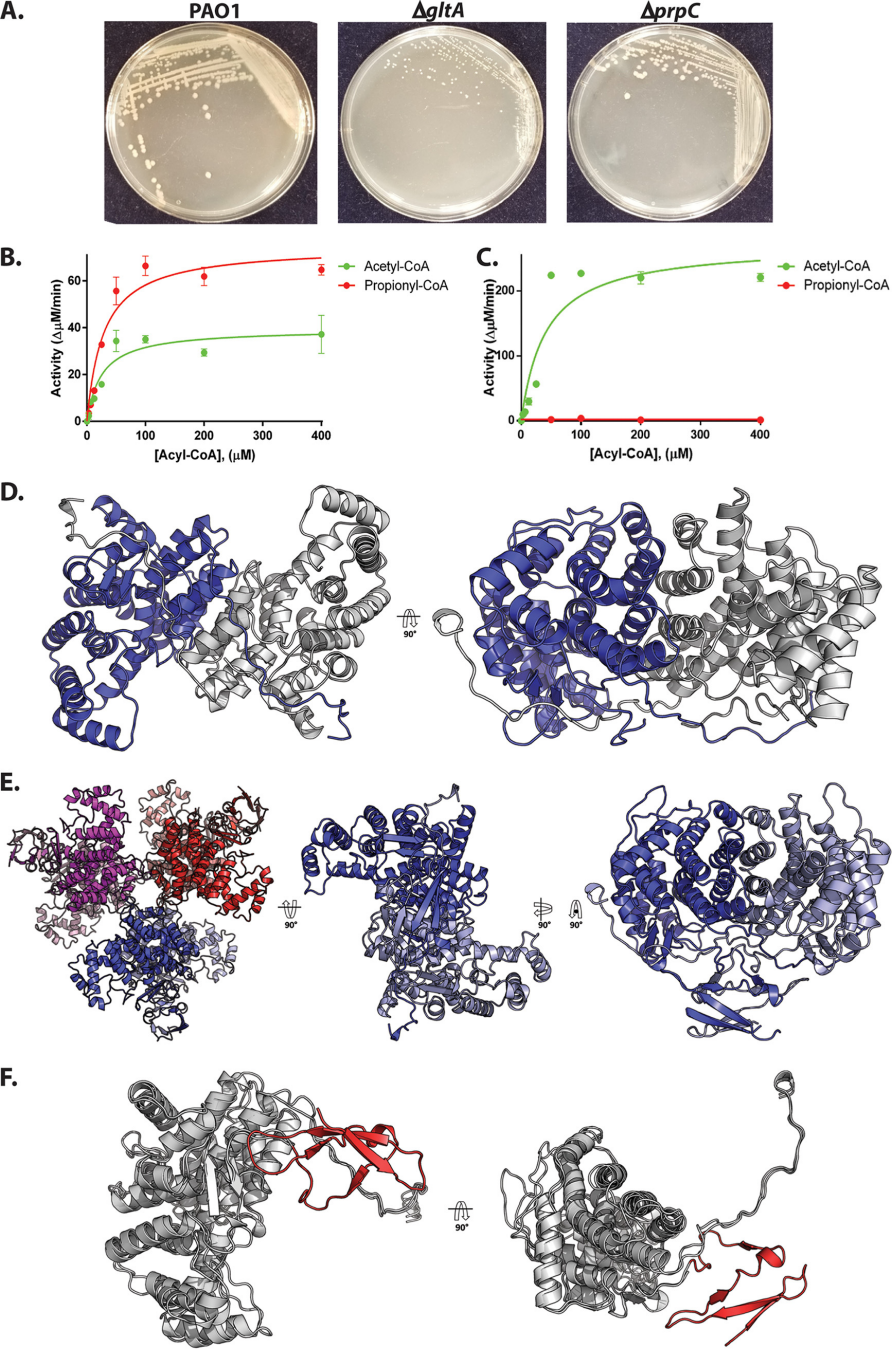
Biochemical and structural analysis of PrpC and GltA from Pa. (A) A Δ*gltA* mutant exhibits a growth defect when cultured on LB agar, whereas a Δ*prpC* mutant displays a wild-type colony morphotype. The plates were photographed after 48 h. The data are representative of two independent experiments, each performed in triplicate. (B) Purified PrpC_Pa_ exhibits both citrate synthase activity (with acetyl-CoA as a substrate) and 2-methylcitrate synthase activity (with propionyl-CoA as a substrate). The concentration of OAA in each reaction was fixed at 0.5 mM. The data are representative of two independent experiments, each performed in triplicate. (C) Purified GltA_Pa_ is a citrate synthase with no detectable 2-methylcitrate synthase activity. The concentration of OAA was fixed at 0.5 mM. The data are representative of two independent experiments, each performed in triplicate. (D) The X-ray crystal structure of PrpC_Pa_ (PDB: 6S6F). PrpC_Pa_ is a homodimer. In the ribbon diagram shown, the protomers are colored blue and gray. (E) Cartoon representation of the GltA_Pa_ hexamer in the asymmetric unit (left) and, for comparison with PrpC_Pa_, the extracted GltA_Pa_ dimers (middle and right). (F) Superposition of the PrpC_Pa_ and GltA_Pa_ structures. PrpC_Pa_ and GltA_Pa_ share similar core α-helical folds (shown in gray to highlight similarities). However, GltA_Pa_ has an additional antiparallel β-sheet at its N terminus (colored in red to showcase differences).

PrpC catalyzes the condensation of oxaloacetate and propionyl-CoA. In a parallel reaction, the TCA cycle enzyme citrate synthase (GltA) catalyzes the condensation of oxaloacetate and acetyl-CoA. Given the apparent promiscuity of AcsA with respect to acetate and propionate activation, we wondered whether the condensation of propionyl-CoA and acetyl-CoA with oxaloacetate could be carried out interchangeably by PrpC and GltA (Fig. 3D). Indeed, PrpC from E. coli has secondary citrate synthase activity, and overexpression of *prpC* in this organism can rescue the synthetic lethality of citrate synthase loss (42–45). To examine whether this is also the case in Pa, a Δ*gltA* mutant was generated. Colonies of the Δ*gltA* mutant on LB agar were visibly smaller than wild-type PAO1 (Fig. 4A). This phenotype could be partially complemented by supplementing the plates with glutamate, whose carbon skeleton enters the TCA cycle after the citrate synthase-catalyzed step (Fig. S4A).

10.1128/mbio.02541-22.4 FIG S4 Supplementary data for structural analysis. (A) The growth defect leading to a small colony size in the Δ*gltA* mutant is partially complemented by supplementing the LB agar with additional glutamate (5 mM). The data represent two independent experiments, each performed in triplicate. (B) Oxaloacetate dependence of the 2-methylcitrate synthase activity of PrpC_Pa_. The concentration of propionyl-CoA was fixed at 0.5 mM. The data represent two independent experiments, each performed in triplicate. (C) Oxaloacetate (200 μM) binding to PrpC_Pa_ leads to a 10°C increase in the melting temperature of the enzyme (*P* < 0.0001, two-tailed unpaired Student’s *t* test). The data represent two independent experiments, each performed in triplicate. (D) Determination of PrpC_Pa_ (1 mg mL^−1^) oligomeric state by analytical ultracentrifugation sedimentation velocity. One hundred and twenty-eight absorbance scans were recorded at 280 nm. Given that the theoretical molecular mass of a PrpC_Pa_ monomer is 41.7 kDa, the single peak of 79.9 kDa suggests that PrpC_Pa_ likely forms a dimer in solution. The calculated frictional coefficient of 1.2 indicates that PrpC_PA_ is likely globular. (E) Alignment of secondary structural elements in a PrpC_Pa_ protomer against other PrpC structures available on the PDB, Coxiella burnetii, S. enterica (Typhimurium), and Mycobacterium tuberculosis (PDB entries 3TQG, 3O8J, and 3HWK). Most of the structures have moderate to high amino acid sequence identity (40 to 60%) with PrpC_Pa_. The structure similarity analysis tool, PDBeFold, revealed 95% secondary structural identity with an RMSD of 1.5 Å, indicating that PrpC is structurally conserved across these bacterial species. Eukaryotic PrpC (from Aspergillus fumigatus, sharing only 24% sequence identity with PrpC_Pa_) has an additional 50 amino acid residues that form an extra loop and helix at the N terminus. Download FIG S4, TIF file, 2.4 MB. Copyright © 2022 Dolan et al. 2022 Dolan et al. https://creativecommons.org/licenses/by/4.0/ This content is distributed under the terms of the Creative Commons Attribution 4.0 International license.

To assess directly whether PrpC_Pa_ has citrate synthase activity (and whether citrate synthase may also have 2-MC synthase activity), we purified each enzyme to investigate its specificity and kinetic properties *in vitro*. The Pa *prpC* and *gltA* genes were cloned and overexpressed (with cleavable His_6_ tags) in E. coli and purified to homogeneity. Each purified enzyme was then assayed for 2-methylcitrate synthase activity and citrate synthase activity. The PrpC enzymes from species including S. enterica, E. coli, and Bacillus subtilis have previously been reported to exhibit a strong preference for propionyl-CoA compared with acetyl-CoA (44, 46, 47). However, PrpC_Pa_ displayed roughly comparable activity toward these acyl-CoAs, although *V*_max_ was greater with propionyl-CoA as a substrate (Fig. 4B; Fig. S4B). The specificity (expressed as *k*_cat_/*K_m_*) of PrpC_Pa_ for propionyl-CoA was 104 × 10^3^ M^−1^ s^−1^, whereas for acetyl-CoA, *k*_cat_/*K_m_* was 114 × 10^3^ M^−1^ s^−1^ (Table S1C). By contrast, and unlike GltA from S. enterica (which exhibits a low level of 2-methylcitrate synthase activity [48]), GltA from Pa (GltA_Pa_) had no detectable 2-methylcitrate synthase activity (Fig. 4C).

10.1128/mbio.02541-22.7 TABLE S1 (A) Oligonucleotide primers used in this study. (B) Bacterial strains and plasmids used in this study (73, 84, 103, 104). (C) PrpC kinetic parameters. (D) Crystallographic statistics for the PrpC-apo, PrpC-oxaloacetate bound, and GltA-apo structures. Download Table S1, DOCX file, 0.1 MB. Copyright © 2022 Dolan et al. 2022 Dolan et al. https://creativecommons.org/licenses/by/4.0/ This content is distributed under the terms of the Creative Commons Attribution 4.0 International license.

**FIG 5:**
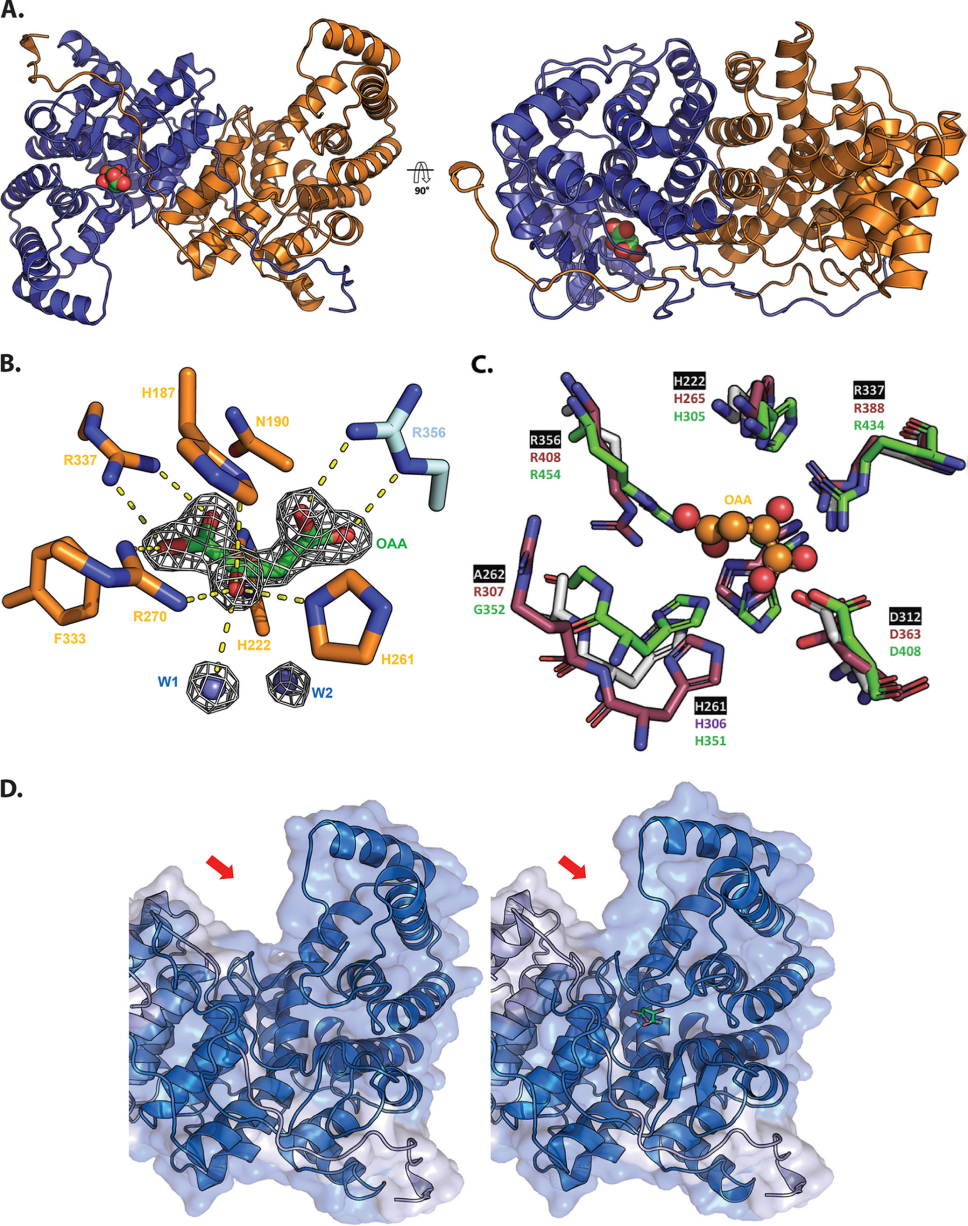
Structural analysis of oxaloacetate-bound PrpC_Pa_. (A) Crystal structure of oxaloacetate-bound PrpC_Pa_ represented in cartoon (PDB: 6S87). One protomer is colored blue and the other orange. A 90° rotation about the *x* axis is shown (right). Oxaloacetate is shown as green and red spheres. (B) Oxaloacetate binding site from P. aeruginosa PrpC chain D. Water molecules are shown in cyan spheres. Chain D and chain C residues are shown in orange and cyan, respectively. The electron density map (2*F_o_-F_c_*) in white is contoured at 1.5σ. (C) Superposition of the PrpC_Pa_ (white), GltA_Pa_ (pink), and A. fumigatus PrpC (5UQR) (green) oxaloacetate binding site. Oxaloacetate is shown in orange spheres. Most of the amino acid residues forming this site are conserved, except R307 (GltA_Pa_ numbering). (D) The left-hand image shows the open (red) apo conformation of PrpC_Pa_, the middle image shows the partially closed (blue) holo conformation of PrpC_Pa_, and the right-hand image shows a superposition of both conformations of PrpC_Pa_. Note the structural rearrangement in the oxaloacetate-bound PrpC_Pa_ protomer (indicated by the red arrow).

To gain insights into the possible structural bases for these kinetic data, we used X-ray crystallography to solve the structure of PrpC_Pa_ and GltA_Pa_ (Fig. 4D). PrpC_Pa_ is a homodimer in both the crystal structure (Fig. 4D) and in solution (Fig. S4D), whereas the GltA_Pa_ asymmetric unit was comprised of a hexameric “trimer of dimers” (Fig. 4E). Structural superposition of PrpC_Pa_ and GltA_Pa_ revealed a near-identical α-helical core fold (Fig. 4F) with a C-α root mean square deviation (RMSD) of 1.33 Å. GltA_Pa_ is slightly larger than PrpC_Pa_ (429 amino acids versus 376 amino acids, respectively) and has an additional 50 amino acid residues at its N terminus, which form four antiparallel β-strands and loops (Fig. 4F). In addition to solving the apo structures of the enzymes, we also obtained the structure of PrpC_Pa_ with oxaloacetate bound in the active site (Fig. 5A). The active site was located in a cleft between two domains on the enzyme. A comparison of PrpC structures from different bacterial species revealed that the residues comprising the PrpC_Pa_ active site are very highly conserved (Fig. S4E). For instance, in S. enterica, His-235 (His-222 in PrpC_Pa_), His-274 (His-261 in PrpC_Pa_), and Asp-325 (Asp-312 in PrpC_Pa_) form a catalytic triad (47).

In the PrpC_Pa_ apo structure (open conformation), each protomer in the asymmetric unit is identical (backbone RMSD of 0.23 Å from a total of 360 C-α atoms). However, in the holo-PrpC_Pa_ structure, the conformation of one of the oxaloacetate-bound protomers (chain D) in the asymmetric unit was different. Each asymmetric unit comprised four monomers of PrpC_Pa_, but only chain D contained an unambiguous electron density for oxaloacetate (Fig. 5B). The other chains (chains A, B, and C) had an identical conformation to those of apo-PrpC_Pa_. Interestingly, the dimerization partner of chain D, chain C, (shown in orange in Fig. 5A) had no oxaloacetate in its active site. This raises the possibility that PrpC_Pa_ exhibits half of the site reactivity, where only one-half of the identical subunits are active at any given time (49).

GltA_Pa_ has the essential catalytic triad of residues that are also found in the *Sus scrofula* citrate synthase, His-265, His-306, and Asp-363 (Pa numbering). The side chain orientation in this triad is identical in the majority of apo-PrpC and apo-GltA structures, including PrpC_Pa_ (Fig. 5C). In S. enterica PrpC, Tyr-197 and Leu-324 (Tyr-184 and Ala-311 in PrpC_Pa_) have been proposed to confer substrate specificity (47). The corresponding residues in the citrate synthases are histidine and valine (His-227 and Val-363 in GltA_Pa_). However, the PrpC from A. fumigatus also has histidine and valine in these positions; hence, the precise role(s) of these residues in imparting substrate specificity are still not clear. In addition to binding its substrate, citrate synthase from E. coli also binds NADH and may even be regulated by this compound. The residues important for NADH binding in GltA from E. coli are Met-112 and Cys-206 (46). These residues are also present in GltA_Pa_, but they are absent from PrpC_Pa_. This is consistent with the notion that GltA_Pa_ is probably regulated by NADH (50), whereas this is probably not the case for PrpC_Pa_.

Superposition of the apo-PrpC_Pa_ and holo-PrpC_Pa_ structures highlights the conformational change associated with oxaloacetate binding (Fig. 5D). The oxaloacetate-bound PrpC_Pa_ has a more compact configuration, achieved through a 2-Å (average) movement and 7° rotation of the associated domain toward the center of the dimer. This conformation was also observed in the Sus scrofa citrate synthase, where it was described as a “partially closed conformation” (51). The fully closed conformation was observed when both oxaloacetate and acetyl-CoA were bound to the enzyme (52). Compellingly, all acyl-CoA-bound citrate synthase structures in the Protein Data Bank (PDB) contain either bound oxaloacetate or bound citrate. This suggests an ordered reaction sequence. Indeed, in citrate synthase, the binding of oxaloacetate has been biochemically and structurally demonstrated to bring about a conformational change, which appears to be critical for the subsequent binding of acetyl-CoA (51). Presumably, a similar ordered reaction sequence is associated with PrpC_Pa_. Consistent with the notion that oxaloacetate binding is accompanied by a conformational change in the enzyme, we observed increased thermal stability of PrpC_Pa_ after the addition of oxaloacetate (Fig. S4C).

### Transcriptomics reveals how Pa responds to challenge with exogenous propionate.

**FIG 6:**
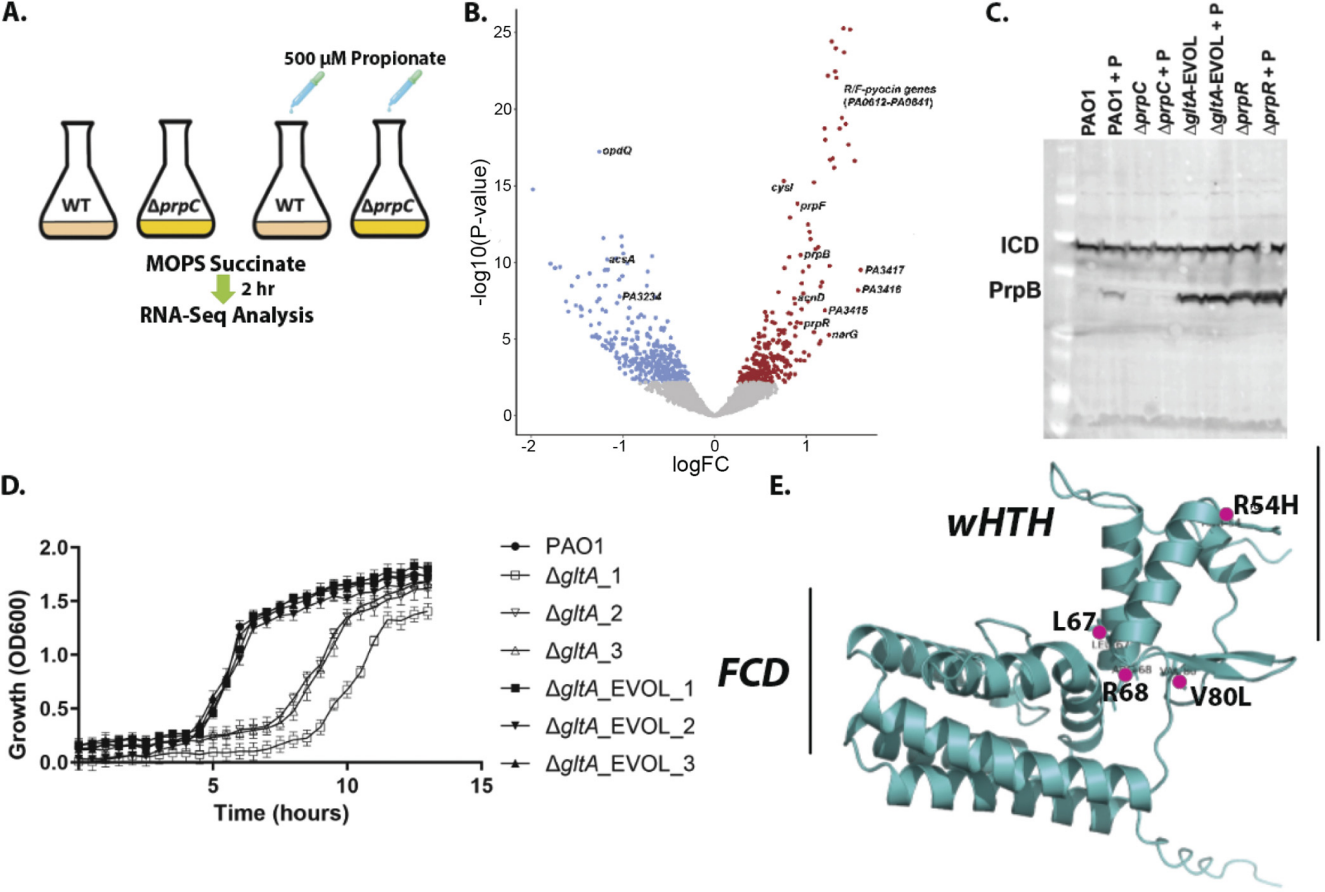
RNA-seq analysis uncovers that propionate exposure induces expression of the *prp* operon and of the genes associated with branched-chain amino acid catabolism in Pa. (A) Schematic of the experimental design. At an OD of 0.2, 500 μM sodium propionate was spiked into (triplicate) cultures of PAO1 and the Δ*prpC* mutant. An equal volume of H_2_O was added to the control PAO1 and Δ*prpC*-mutant cultures (also grown in triplicate). The cultures were harvested 2 h after the propionate addition, corresponding to an OD_600_ of ≅0.6 (exponential growth), and RNA-seq analysis was carried out. (B) Volcano plot illustrating the log_2_ fold change in transcript abundance versus adjusted *P* values for wild-type PAO1 grown in MOPS-succinate versus wild-type PAO1 grown in MOPS-succinate + 500 μM propionate. Transcripts that are significantly (*q* < 0.05) increased (red) or decreased (blue) in abundance are indicated. Selected transcripts are labeled. (C) Western blot showing protein expression levels of PrpB (32.1 kDa) in PAO1 and in the Δ*prpC* mutant, the Δ*gltA*_EVOL mutant, and the Δ*prpR* mutant after exposure to 4 mM propionate (+P) for 3 h. Isocitrate dehydrogenase (ICD; 45.6 kDa) served as loading control. Note that the Δ*gltA*_EVOL and Δ*prpR* mutants display constitutively active PrpB expression, independent of propionate addition. Data are representative of three independent experiments. (D) Growth of Δ*gltA* and Δ*gltA_*EVOL1 to 3 mutants compared with PAO1 in MOPS-acetate medium. The data are representative of three independent experiments, each performed in triplicate. (E) AlphaFold model of PrpR with the locations of the residues mutated and/or deleted in the Δ*gltA_*EVOL_1 to 3 mutants highlighted. The winged helix-turn-helix (wHTH) motif and the GntR family FadR C-terminal domain (FCD) are shown.

From the proteomic data, PrpC_Pa_ (and all other enzymes of the 2MCC) were detectable during growth of Pa on succinate as a sole carbon source, so it is possible that the 2MCC also serves a noncanonical, uncharacterised role(s) in Pa physiology. To explore this further, RNA sequencing (RNA-seq) was used to (i) compare the transcriptome of wild-type PAO1 with that of an isogenic Δ*prpC* mutant during growth on succinate and (ii) examine how the transcriptome is perturbed following exposure of succinate-grown cells to a subinhibitory concentration (500 μM) of propionate (added during exponential growth [Fig. 6A; Fig. S5A to E]).

10.1128/mbio.02541-22.5 FIG S5 Transcriptomic analysis (DESeq2) of Pa wild type and the Δ*prpC* mutant grown on MOPS-succinate versus MOPS-succinate containing 500 μM propionate for 2 h. (A) A principal component(s) analysis (PCA) scores plot of the RNA-seq replicates (triplicates). Wild-type cultured in MOPS-succinate (blue), wild-type cultured in MOPS-succinate + propionate (red), Δ*prpC* mutant cultured in MOPS-succinate (purple), and Δ*prpC* mutant cultured in MOPS-succinate + propionate (green) are shown. (B) Sequencing (transcript) reads corresponding to the 2MCC operon for PAO1 and the Δ*prpC* mutant ± propionate. The *prpC* transcript reads are absent from the Δ*prpC* mutant with no obvious polar effects on the downstream ORFs. (C) Volcano plot illustrating the log_2_ fold change in transcript abundance versus adjusted *P* values for the Δ*prpC* mutant grown in MOPS-succinate versus the same mutant grown in MOPS-succinate + 500 μM propionate. Transcripts that are significantly (*q* < 0.05) increased (red) or decreased (blue) in abundance are indicated. Selected transcripts are labelled. (D) Volcano plot illustrating the log_2_ fold change in transcript abundance versus the adjusted *P* value for the Δ*prpC* mutant grown in MOPS-succinate medium compared with the wild-type grown in the same medium. The transcripts that are significantly (*q* < 0.05) increased (red) or decreased (blue) in abundance are indicated. Notable transcripts are labeled (*bkdA1*, *cysI*, and *cysD*). (E) Volcano plot illustrating protein log_2_ fold change in transcript abundance versus adjusted *P* value for the Δ*prpC* mutant grown in MOPS-succinate + propionate versus the wild-type grown in the same medium. Transcripts that are significantly (*q* < 0.05) increased (red) or decreased (blue) in abundance are indicated. Notable ORFs are labeled (PA3619, *prpB*, *metE*, PA2603, *prpR*, *prpF*, *acnD*, and PA3391). (F) Western blot showing PrpB (32.1 kDa) protein expression levels in PAO1, the Δ*prpC* mutant, and the Δ*acsA* mutant after exposure to increasing concentrations (0 mM, 2 mM, and 4 mM) of propionate for 3 h. Isocitrate dehydrogenase (ICD; 45.6 kDa) served as a loading control. Data are representative of three independent experiments. (G to I) Growth of the wild type (PAO1), a Δ*prpR* mutant, a Δ*crc* mutant, a Δ*cbrB* mutant, a Δ*acsA* mutant, and a Δ*erdR* mutant in MOPS-succinate (G), MOPS-propionate (H), and MOPS-acetate (I) media. The data are representative of three independent experiments, each performed in triplicate. (J) Western blot showing PrpB and ICD expression levels in the indicated mutants (Δ*prpC*, Δ*crc*, Δ*cbrB*, Δ*pycA*, and Δ*erdR*) grown in the presence and absence (as indicated) of propionate. Data are representative of two independent experiments. Download FIG S5, PNG file, 0.9 MB. Copyright © 2022 Dolan et al. 2022 Dolan et al. https://creativecommons.org/licenses/by/4.0/ This content is distributed under the terms of the Creative Commons Attribution 4.0 International license.

Consistent with the proteomic data, appreciable *prpC* reads were detected in PAO1 during growth on succinate (Fig. S5B). This basal expression of the 2MCC enzymes may benefit the cell by priming it ready for rapid propionyl-CoA detoxification/catabolism. Relatively few PAO1 transcripts showed substantial alterations in abundance compared with the Δ*prpC* mutant during growth on MOPS-succinate medium (Data Set S3; Fig. S5D). This suggests that the absence of a functional 2MCC does not lead to extensive transcriptional reprogramming during *per se*. Transcripts encoding two enzymes (BkdA1 and BkdA2) involved in branched-chain amino acid (BCAA) catabolism were downregulated in the Δ*prpC* mutant compared with the wild-type during growth on succinate. However, this repression was relieved after exposure of the Δ*prpC* mutant to exogenous propionate (2.2 FC). These data indicate that in the wild-type, flux through the 2MCC during growth on succinate may produce low levels of propionate and that this impacts BCAA catabolic gene expression. The source of this propionate could be from the catabolism of endogenously produced propionyl-CoA-generating amino acids or possibly through reverse operation of the 2MCC. The 2MCC has recently to be shown to be reversible in M. tuberculosis to allow optimal metabolism of lactate and pyruvate (53).

10.1128/mbio.02541-22.10 DATA SET S3 Full transcriptomic data of P. aeruginosa PAO1 and Δ*prpC* cultured in MOPS succinate with/without propionate supplementation. Download Data Set S3, XLSX file, 3.3 MB. Copyright © 2022 Dolan et al. 2022 Dolan et al. https://creativecommons.org/licenses/by/4.0/ This content is distributed under the terms of the Creative Commons Attribution 4.0 International license.

The most statistically significant upregulated transcripts in the wild-type following challenge with propionate were associated with ORFs PA3415 to PA3417 (2.8 FC). These ORFs are predicted to encode a pyruvate dehydrogenase (PDH) or a branched-chain amino acid dehydrogenase (Data Set S3; Fig. 6B) (54). The same ORFs were also upregulated in the Δ*prpC* mutant after propionate addition, indicating that full catabolism of propionate is not required as a cue to activate the expression of these genes (Data Set S3; Fig. S5C). Immediately adjacent to the PA3415 to PA3417 cluster is leucine dehydrogenase (*ldh*, PA3418), required for BCAA catabolism, which was also significantly upregulated after propionate exposure (~3-fold change). However, given that propionyl-CoA is an intermediate in the metabolism of BCAA, these results likely indicate regulatory cross-talk between expression of the PA3415 to PA3417-*ldh* cluster and expression of the enzymes involved in BCAA catabolism. Unexpectedly, *acsA* and PA3233 to PA3235 (the ORF cluster that includes the ActP protein), which have putative roles, respectively, in propionate activation and transport, were downregulated (−2.0 FC) in PAO1 after propionate addition.

Pa responds to propionate exposure by increasing expression of the *prp* operon (Fig. 6B). This upregulation of the *prp* operon was blocked in the Δ*prpC* mutant (Fig. S5E; Data Set S3). We further confirmed the induction of PrpB expression in response to propionate by Western blotting (Fig. S5F). However, this propionate-induced expression of PrpB was abolished in the Δ*prpC* mutant (Fig. S5F). PrpB expression in response to propionate challenge was maintained in a Δ*acsA* mutant, suggesting that propionyl-CoA can also be generated from propionate through alternative routes in Pa. *acsA* expression is known to be under the control of the response regulator ErdR, and consistent with this, an Δ*erdR* mutant cannot grow on ethanol or acetate as a sole carbon source (55). Given the dual role that AcsA seems to play in acetate and propionate catabolism, we therefore examined whether a Δ*erdR* mutant also displays aberrant growth on propionate; it did (Fig. S5G to I). Surprisingly, the growth deficit of the Δ*erdR* mutant on propionate was even more pronounced than that of a Δ*acsA* mutant. This indicates that additional downstream targets of ErdR, such as ErcS, ErbR, or the ethanol oxidation system, may also be required for optimal propionate catabolism in Pa (56).

How is propionate assimilated in Pa and converted to propionyl-CoA, activating the 2MCC operon even when preferable carbon sources are readily available? Carbon catabolite repression (CCR) allows Pa to selectively assimilate a preferred compound when a selection of carbon sources is available. In Pa, CCR is controlled through translational silencing, mediated by Hfq and the small protein Crc (57). Reversing this translational silencing requires the small RNA (sRNA) CrcZ, which sequesters Hfq, thereby preventing the latter from binding to target transcripts. CrcZ abundance is controlled by a two-component system, CbrAB, which senses and responds to carbon availability (57). In Pa, *acsA* mRNA harbors a sequence motif located upstream of the *acsA* start codon, which brings acetate assimilation under CCR control. Because they are impaired in CrcZ expression, mutants defective in *cbrB* exhibit a severe growth defect when grown on acetate as a sole carbon source (58) (Fig. S5I). We found that a Δ*cbrB* mutant also had a clear growth defect on propionate (Fig. S5H). However, since the Δ*cbrB* mutant maintained inducible PrpB expression after propionate exposure (Fig. S5J), this suggests that CCR does not exert direct control over the 2MCC but may impact propionate catabolism indirectly, for example, by affecting *acsA* expression and/or other peripheral targets.

Because CCR did not appear to be directly coordinating the expression of the 2MCC, this prompted us to examine in more detail the role of the GntR-family TF PrpR (PA0797) in controlling *prp* gene expression. GntR family TFs are typically regulated by ligands that are metabolic substrates/products/cofactors associated with the products of the genes that they regulate (59). Previous studies in Corynebacterium glutamicum and S. enterica established that 2-methylcitrate (2-MC), the reaction product of PrpC, is a coactivator of the Fis-family PrpR in these bacteria (60, 61). By contrast, PrpR from M. tuberculosis is a 4Fe4S protein that uses propionyl-CoA as a coactivator (62). Our observation that the 2MCC is not induced after exposure to propionate in a Δ*prpC* mutant (Fig. S5E and F) is consistent with 2-MC rather that propionyl-CoA being the coactivator, especially given that propionyl-CoA accumulates in a Δ*prpC* mutant following propionate challenge (Fig. 3C).

In contrast to all other species characterized to date, the Δ*prpR* mutant of Pa had no growth defect on any of the carbon sources tested (Fig. S5H to J). This suggested that the canonical model of 2MCC regulation by PrpR established for other organisms does not apply in Pa. Remarkably, PrpB was overexpressed in the Δ*prpR* mutant during growth on succinate, independent of the presence or absence of propionate in the medium (Fig. 6C). This suggested that Pa PrpR may actually be a repressor of the 2MCC rather than an activator (60). Consistent with this, expression of *prpR* from a plasmid (pUCP20) in the Δ*prpR* mutant was sufficient to repress PrpC expression in this strain (Fig. S6A). The predicted PrpR binding motif in Pa, identified by phylogenetic footprinting, is a 12-nucleotide palindrome with the consensus sequence ATTGTCGACAAT (16); this sequence is found upstream of PrpR (84 bp) in PAO1 and PA14. PrpR was recombinantly expressed and purified to homogeneity (Fig. S6B) for electrophoretic mobility shift analyses (EMSAs; Fig. S6C). These data revealed that PrpR does indeed bind to the upstream region of *prpR*.

10.1128/mbio.02541-22.6 FIG S6 PrpR functional elucidation and genomic sequencing of the “evolved” Δ*gltA* mutants Δ*gltA*_EVOL_1 to 3. (A) Western blot demonstrating that complementation of the Δ*prpR* mutant and the Δ*gltA_*EVOL mutant with a plasmid overexpressing *prpR* (pUCP20_*prpR*) reverts PrpC expression to levels seen in the wild type in the absence of added propionate. Note that introduction of an empty vector (pUCP20_CTL) into these mutants does not affect PrpC expression compared with the corresponding vector-less mutants. The cultures were all grown in LB medium. Isocitrate dehydrogenase (ICD) served as a loading control. The data are representative of three independent experiments. (B) SDS-PAGE analysis showing recombinant expression and purification of His_6_-PrpR (≈27 kDa) in E. coli. Lanes 1 to 4 show eluted protein fractions from Ni-NTA affinity chromatography; lane 4, nickel-column flow through; lane 5, whole-cell lysate. (C) Electrophoretic mobility shift assay (EMSA) polyacrylamide gel showing binding of recombinant PrpR to a PCR-amplified segment of DNA containing the *prpR* promoter labeled with the 6-FAM (5 pM), resulting in a protein-DNA complex (lanes 5 to 7). Lanes 1 to 6 show increasing PrpR concentration: 0.06 μM, 0.125 μM, 0.25 μM, 0.5 μM, 1 μM, and 2 μM. Lane 7 shows nonspecific competitor (*ccoN1* promoter) in the presence of 2 μM PrpR, and lane 8 shows an unlabeled competitor (the PCR amplicon containing the *prpR* promoter but not the 6-FAM-labeled promoter) in the presence of 2 μM PrpR; NEG, negative control (no PrpR added). The data are representative of three independent experiments. (D) Growth of PAO1 compared with the Δ*gltA*, Δ*prpR* Δ*gltA*, and Δ*gltA*_EVOL mutants in LB medium. The data are representative of three independent experiments, each performed in triplicate. (E) Integrative Genomics Viewer (IGV) track highlighting the *gltA* (top) and *prpR*-*prpC* (bottom) genomic regions in wild-type PAO1 and in the Δ*gltA_*EVOL_1 to 3 mutants. Reads corresponding to *gltA* are absent in the Δ*gltA_*EVOL_1 to 3 mutants (as expected), and the single nucleotide polymorphisms and deletions in *prpR* are highlighted (between the red dashed lines). Download FIG S6, PNG file, 1.6 MB. Copyright © 2022 Dolan et al. 2022 Dolan et al. https://creativecommons.org/licenses/by/4.0/ This content is distributed under the terms of the Creative Commons Attribution 4.0 International license.

Somewhat surprisingly, we found that a Pa citrate synthase mutant (Δ*gltA*; Fig. 4A) could also grow on single carbon sources in minimal medium, albeit with a prolonged lag phase. Given that PrpC_Pa_ can carry out the condensation of acetyl-CoA with oxaloacetate (Fig. 4B) and can therefore potentially substitute for GltA, we suspected that the viability of the Δ*gltA* mutant on single carbon sources might be explained by induction of *prpC*. Consistent with this, and despite repeated attempts at doing so, we were unable to make a Δ*gltA* Δ*prpC* double mutant. If our hypothesis is correct, and given that *prpC* expression is repressed by PrpR, we began to wonder whether *prpR* in the Δ*gltA* mutant might be under strong selection pressure to acquire loss-of-function mutations, thereby boosting PrpC expression. Commensurate with this, successive rounds of subculturing of the Δ*gltA* mutant in MOPS-succinate readily yielded heritable derivatives displaying a restoration of rapid growth in this medium. Furthermore, these “evolved” Δ*gltA* mutants also constitutively expressed PrpC and PrpB independent of propionate addition (Fig. 6C; Fig. S6A). A similar restoration of rapid growth in MOPS-succinate medium was observed when we deleted *prpR* in the Δ*gltA* background (Fig. S6D).

But are loss-of-function mutations in *prpR* the most probable evolutionary path taken by the Δ*gltA* mutant to overcome its metabolic bottleneck? Alternative mechanisms could include mutation of the PrpR binding site upstream of the *prp* operon, duplication of *prpC*, or inactivation of one or more uncharacterized genes involved in modulating PrpR expression. To investigate this further, we made fresh deletions in *gltA* (to minimize the possibility of the strain acquiring additional mutations before passaging). Three independent Δ*gltA*-mutant colonies were isolated and passaged in MOPS-succinate for 2 days; all three cultures displayed wild-type levels of growth after this time (Fig. 6D; Fig. S6D). The three independently evolved Δ*gltA* mutants were sent for whole-genome sequencing alongside the wild-type progenitor (Fig. S6E). Strikingly, each of the three evolved Δ*gltA* mutants (EVOL_1 to EVOL_3) had accrued distinct missense mutations in *prpR*, giving rise to the amino acid substitutions V80L (EVOL_1) and R54H (EVOL_3) in PrpR or a 6-bp deletion in *prpR* leading to the loss of amino acids L67 and R68 in PrpR (EVOL_2). These residues were mapped on to the AlphaFold-generated structure of PrpR, which indicated that they fall within and proximal to the conserved winged helix-turn-helix (wHTH) domain of this repressor, a region crucial for the DNA binding in this family of transcription factors (Fig. 6E) (63, 64). Indeed, mutation of residue R52 (equivalent to residue R54 in PqsR) in the E. coli MqsR-controlled colanic acid and biofilm regulator (McbR) results in a loss of DNA binding (59). This provides clear evidence that in the absence of *gltA*, *prpR* is reproducibly mutated to facilitate *prpC* overexpression.

## DISCUSSION

**FIG 7:**
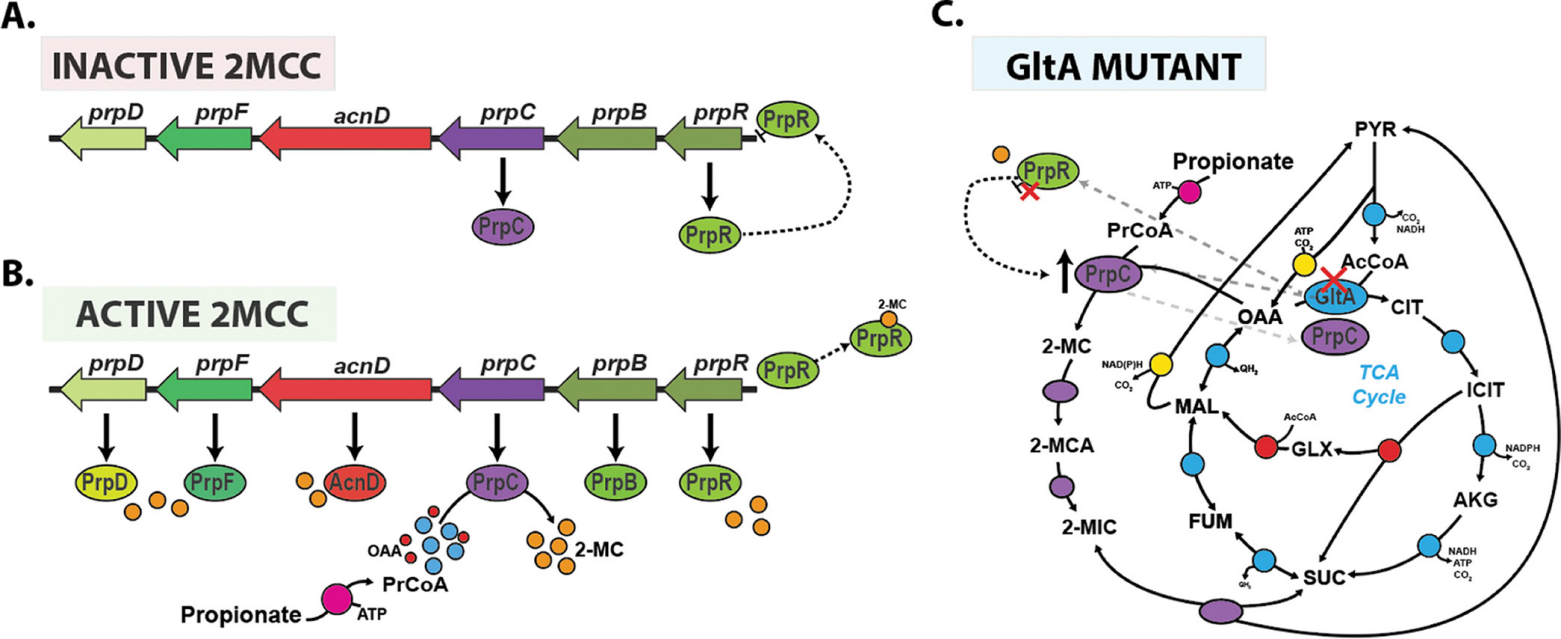
Model for the operation of the 2MCC in Pa. (A) During growth in the absence of propionate or propionyl-CoA generating substrates, the 2MCC operon (*prp*) expression is repressed through the binding of PrpR to its upstream promoter region. Incomplete repression of the operon (from basal cellular propionyl-CoA or competing transcriptional activators) results in a basal, low level of *prpC* transcription. (B) As the cellular propionyl-CoA levels rise, this metabolite is condensed with oxaloacetate by PrpC, resulting in the formation of 2-MC; 2-MC likely then binds to PrpR, inducing conformational changes that lead to the dissociation of PrpR from the DNA. This derepresses the *prp* operon, allowing expression of the 2MCC enzymes. However, as the concentration of propionyl-CoA falls (due to depletion of propionate or BCAAs due to 2MCC activity) so too does the concentration of 2-MC, which, in turn, leads to rebinding of PrpR to the *prp* promoter region and a resumption in *prp* operon repression. (C) In the absence of citrate synthase (GltA), Pa can survive because of the low-level basal expression of PrpC, a promiscuous enzyme that also has citrate synthase activity. However, this low total citrate synthase activity is unable to meet cellular demand, resulting in a severe growth defect and a strong selection pressure to acquire mutations that increase *prpC* expression. Based on our work, it seems that mutations in *prpR* that abolish its repressor activity are the most commonly selected mechanism for achieving this. These mutations lead to constitutive expression of the *prp* genes and, thus, an increase total cellular citrate synthase activity (compensating for the loss of GltA activity).

We have carried out a systems-level characterization of Pa during growth on succinate and propionate as sole carbon sources. This revealed previously undiscovered transcriptional and metabolic cross-talk between several major metabolic pathways/cycles: the 2MCC, BCAA catabolism, and the glyoxylate shunt. Our work also provides mechanistic insight into how enzyme promiscuity and regulatory rewiring can rapidly overcome the loss of a key enzyme in the TCA cycle, citrate synthase. We show that Pa can survive the loss of citrate synthase (GltA) through a combination of low, basal-level expression of PrpC, followed by acquisition of loss-of-function mutations in the transcriptional repressor *prpR*. This leads to a compensatory increase in secondary citrate synthase activity through PrpC overexpression (Fig. 7A to C).

We found that Pa responds to propionate exposure by increasing expression of the *prp* operon. This propionate-dependent expression of the 2MCC was unaffected by carbon catabolite repression (CCR) or by deletion of the primary short-chain acyl-CoA synthetase AcsA. This may reflect the established appetite of Pa for organic acids (1), but it could also be that the primary role of the 2MCC is in propionate detoxification rather than routine carbon assimilation. It appears that Pa counters rapid propionyl-CoA generation by having an exceptionally responsive 2MCC, which promptly degrades inhibitory metabolic intermediates. In M. tuberculosis, this detoxification is carried out by the constitutively expressed methylmalonyl-CoA (MMCO) pathway, which can quickly react to sudden changes in propionate concentration and detoxify the cell accordingly. By contrast, the role of the M. tuberculosis 2MCC appears to be as a “professional catabolizer,” with a higher overall flux capacity than the MMCO (65). The absence of a functional MMCO in Pa means that this organism depends exclusively on the 2MCC for both the assimilation and detoxification of propionate.

The trade-off between responsiveness to propionate and the accumulation of cytotoxic 2MCC intermediates is a structural weakness of this catabolic arrangement, a weakness that can potentially be exploited to fight Pa infections. Importantly, a synthetic PrpC inhibitor was bacteriostatic against M. tuberculosis grown in cholesterol medium (cholesterol is broken down by M. tuberculosis to yield propionyl-CoA). This suggests that cell-permeable PrpC-specific inhibitors are indeed achievable (66). Considering the structural similarity between PrpC and GltA, it may be possible to generate an inhibitor, which targets both enzymes simultaneously. This could be a powerful combination, as a transposon mutagenesis screen indicated that *gltA* is required for the growth of nine different Pa strains from diverse sources when cultured under four infection-relevant growth conditions (LB, M9 glucose, sputum, and serum) (67). Interestingly, most of these strains did not require *gltA* for growth in urine (67, 68). However, the current work highlights the risk of targeting GltA exclusively, since Pa can swiftly compensate for the loss of GltA activity by increasing PrpC expression. This can be accomplished through PrpR inactivation, either via the acquisition of a loss-of-function mutation in *prpR* or through derepression. The latter requires a low basal level of PrpC expression to generate the requisite 2-MC (Fig. 7).

Mutations in core metabolic genes are strongly associated with antimicrobial resistance, although our insight of the mechanistic basis for this is poorly understood (69–71). Crucially, pathogen lifestyles vary, and this in turn leads to major alterations in the regulatory architecture of primary metabolism. These design variations mean that many of the metabolic innovations that facilitate adaptation to new environments (or to antimicrobial challenge) are pathogen specific.

Can we predict the potential routes of mutation and genetic evolution? Addressing this is a central challenge for evolutionary systems biology and requires a clear understanding and appreciation of microbial metabolic network diversity. As shown in the current work, large-scale comparative ‘omics analyses, in combination with reverse genetics, can provide mechanistic insights into the complex evolutionary trajectories of underground metabolism. Indeed, the specific path to derepression of *prpC* expression in Pa (via *prpR* inactivation) that allows the cell to survive in the absence of citrate synthase simply cannot happen in E. coli or indeed in many other human pathogens due to key differences in metabolic architecture, enzymology, and gene regulation (72). Therefore, the strategic inhibition of organic acid catabolism in Pa through inhibition of PrpC and GltA activity may be a potent mechanism to halt the growth of this pathogen during infection in environments where propionate is abundant.

## MATERIALS AND METHODS

### Growth conditions.

Unless otherwise indicated, P. aeruginosa strain PAO1 (73) was routinely grown in lysogeny broth (LB; Lennox, Oxoid Ltd.) at 37°C with good aeration (shaking at 250 rpm). The strains used in this study are listed in Table S1A in the supplemental material. The overnight precultures were started from separate clonal source colonies on streaked LB agar plates. Strains were cultured in MOPS (morpholinepropanesulfonic acid) medium with the relevant carbon sources (74). Cell growth was monitored as optical density in a spectrophotometer (BioSpectrometer, Eppendorf) at a wavelength of 600 nm (OD_600_). A previously determined conversion factor of 0.42 g cell dry weight (CDW) per OD_600_ unit was used to calculate biomass specific rates and yields from the obtained OD_600_ values (75).

### Transcriptomics (RNA-seq).

P. aeruginosa strain PAO1 and Δ*prpC* were grown in 40 mL of MOPS with succinate (30 mM) as the sole carbon source (six flasks per strain) at 37°C with good aeration (shaking at 250 rpm) in baffled flasks (500-mL volume). At OD_600_ of 0.2, 500 μM sodium propionate was spiked into three of the PAO1 cultures and three of the Δ*prpC* cultures. An equal volume of H_2_O was added to the control PAO1 and Δ*prpC* cultures. After 2 h, an aliquot (5 mL) of culture was removed from each sample. At this stage, the culture OD_600_ was ≅0.7 (exponential growth). These aliquots were added to an equal volume of RNAlater RNA stabilization solution. RNA was then isolated using a RNeasy minikit (Qiagen). rRNA was subsequently depleted from each RNA sample (5 μg each) using the bacterial Ribo-Zero rRNA removal kit (Illumina). The integrity of the RNA was evaluated using an RNA 6000 Nano LabChip and an Agilent 2100 Bioanalyzer (Agilent Technologies, Germany). Twelve indexed, strand-specific cDNA libraries were prepared, and samples were sequenced on an Illumina HiSeq 2000 with a 51-bp single-end read length (GATC Biotech, Germany).

### Reads mapping and annotations.

The FASTQ files were mapped to the PAO1 genome obtained from the Pseudomonas Genome Database (PGD) (http://www.pseudomonas.com/) using Bowtie v.0.12.8 (38). The sequence reads were adaptor clipped and quality trimmed with Trimmomatic (76) using the default parameters. The Integrative Genomics Viewer (IGV) was used to visually inspect mapping quality and the absence of *prpC* reads in the Δ*prpC* mutant. Read summarization was performed using featureCounts (77). DESeq2 was used to analyze differentially expressed genes (78). Annotations of differentially expressed genes were obtained from the reference annotation of the Pseudomonas genome available at the PGD website. Genes were considered significantly induced or repressed when their adjusted *P* value was <0.05 (Data Set S3).

### Quantitative proteomics analysis.

P. aeruginosa PAO1 cells (OD_600_ = 0.5, 30 mL) were grown at 37°C in 40 mL of MOPS with succinate (30 mM) or propionate (40 mM) as the sole carbon source and good aeration (shaking at 250 rpm) in baffled flasks (500-mL volume). Cultures were grown and analyzed in triplicate. The cell pellets were resuspended in 2 mL of lysis buffer (100 mM Tris-HCl, 50 mM NaCl, 10% [vol/vol] glycerol, and 1 mM *tris*(2-carboxyethyl)phosphine [TCEP], pH 7.5) containing one cOmplete Mini protease inhibitor cocktail (Roche). Following three rounds of sonication (3 × 10 s) on ice, supernatants were clarified by sedimentation (21,130 × *g*, 15 min, 4°C) in an Eppendorf 5424R centrifuge. Aliquots (100 μg) of each sample were reduced with TCEP, alkylated with iodoacetamide, and labeled with tandem mass tags (TMTs). TMT labeling was carried out according to the manufacturer’s protocol.

### Liquid chromatography-tandem mass spectrometry (LC-MS/MS).

LC-MS/MS analyses were carried out using a Dionex Ultimate 3000 RSLC nanoUPLC (Thermo Fisher Scientific, Inc., Waltham, MA, USA) system in-line with a Lumos Orbitrap mass spectrometer (Thermo Fisher Scientific, Inc., Waltham, MA, USA) (27). Separation of peptides was performed by C_18_ reverse-phase chromatography at a flow rate of 300 nL/min using a Thermo Scientific reverse-phase nano EASY-Spray column (Thermo Scientific PepMap C_18_, 2-μm particle size, 100-Å pore size, 75-μm inner diameter [i.d.] × 50-cm length).

### Proteomics data analysis.

Proteome Discoverer v2.1 (Thermo Fisher Scientific) and Mascot (Matrix Science) v2.6 were used to process raw data files. The data were aligned with the UniProt Pseudomonas aeruginosa (5,584 sequences) common repository of adventitious proteins (cRAP) v1.0. The R package MSnbase (79) was used for processing proteomics data. Protein differential abundance was evaluated using the Limma package (80). Differences in protein abundances were statistically determined by Student’s *t*-test, with variances moderated by Limma’s empirical Bayes method. *P* values were adjusted for multiple testing by the Benjamini-Hochberg method (81). Proteins were considered increased or decreased in abundance when their log_2_ fold change values were >1 or <−1, respectively, and their *P* value was <0.05. The mass spectrometry proteomics data have been deposited to the ProteomeXchange Consortium via the PRIDE (55) partner repository with the data set identifier PXD015792.

### Genome sequencing.

Genomic DNA was extracted from PAO1 and three evolved Δ*gltA* mutants (EVOL_1 to EVOL_3) using a GeneJET genomic DNA purification kit following 50 generations of growth in MOPS-succinate. Genome sequencing of all four strains was carried out by MicrobesNG (http://www.microbesng.uk), and the reads were analyzed and displayed using IGV (82).

### Construction of in-frame P. aeruginosa PAO1 deletion mutants.

Flanking regions 800 to 1,000 bp upstream and downstream of the desired genes were PCR amplified. The upstream and downstream regions were then overlapped and cloned into the suicide vector pEX19Gm using Gibson assembly as described previously (83). The resulting deletion plasmid was then introduced into P. aeruginosa by electroporation and selected for on LB plates containing 50 μg/mL gentamicin. Deletion mutants were identified via SacB-mediated sucrose counterselection and confirmed by PCR. Primers used are described in Table S1B.

### Construction of luciferase reporter strains.

Transcriptional reporter constructs were made by fusing the upstream promoter sequences of the indicated genes with the *luxCDABE* cluster using the primers listed in Table S1B. The purified PCR products were digested and directionally ligated into the multiple cloning site of plasmid pUC18T-mini-Tn7T-lux-Gm (84). The mini-Tn7-lux element was introduced into PAO1 (where it integrated into the chromosome) by electroporation along with the helper plasmid pTNS2, as previously described (85). Luciferase and OD_600_ readings were measured using a BMG Labtech FLUOstar Omega microplate reader. Strains were cultured in MOPS medium with the indicated carbon sources (100 μL) in 96-well microplates (Greiner bio-one, flat-bottom, black) covered with gas-permeable imaging seals (4titude, 4ti-0516/96). Luciferase expression was assessed during exponential growth. Growth was measured by taking OD_600_ readings simultaneously with the luminescence readings. Luciferase readings were expressed as relative luminometer units (RLU) normalized to OD_600_ to control for growth rate differences in the selected carbon sources.

### ^13^C fluxomics.

Starter cultures were prepared by inoculating LB medium with a loop of freshly plated PAO1. After 6 h of incubation, 50 μL of cell suspension was transferred to a second culture of MOPS minimal medium containing the desired substrate (see below). Subsequently, exponentially growing cells were used as an inoculum for the main cultures. In the main cultures, PAO1 was grown in 25 mL of minimal medium in baffled shake flasks (250-mL volume) with good aeration (shaking at 200 rpm at 37°C) in an orbital shaker (Aquatron, Infors AG, Switzerland). Under these conditions, the oxygen level is maintained above 80% of saturation (75).

For the second and main cultures, PAO1 was grown in MOPS minimal medium with 40 mM propionate or 30 mM succinate as the sole carbon source (i.e., 120 mM carbon in each case). For ^13^C flux experiments, naturally labeled propionate and succinate was replaced with separate tracers (three for propionate and two for succinate) to maximize data set resolution and to accurately determine substrate uptake. Naturally labeled propionate was substituted with [1,3-^13^C_2_]sodium propionate (99%), [3-^13^C]sodium propionate (99%), and an equimolar mixture of [U-^13^C_3_]sodium propionate (99%) and naturally labeled sodium propionate (Sigma-Aldrich, Poole, Dorset, UK). Naturally labeled succinate was substituted with 99% [1,4-^13^C_2_]sodium succinate, 99% [2,3-^13^C_2_]sodium succinate, or an equimolar molar 1:1 mixture of [U-^13^C_4_]sodium succinate (obtained from Cambridge Isotope Laboratories, Inc., Andover, MA, USA) and naturally labeled sodium succinate.

In cultures incubated with ^13^C-tracer, the inoculum (initial OD of <0.02) was always kept below 1% of the final sampled cell concentration to exclude potential interference of nonlabeled cells on subsequent calculation of flux (86). Mass isotopomer labeling analysis of proteinogenic amino acids, mass isotopomer labeling analysis of cell sugar monomers (glucose, ribose, and glucosamine), and metabolic reaction network and flux calculation were carried out as described previously (20).

### Quantification of substrates and products.

Propionate and succinate as well as organic acids (citric acid, α-ketoglutaric acid, gluconic acid, 2-ketogluconic acid, pyruvic acid, succinic acid, lactic acid, formic acid, fumaric acid, and acetic acid) were quantified in filtered culture supernatants (Costar Spin-X, 0.22 μm) using isocratic high-performance liquid chromatography (Agilent 1260 Infinity series, s HPX-87H column operating at 65°C and a flow rate of 0.5 mL min^−1^) equipped with refractive index (RI) and UV detectors (210 nm) with 12 to 50 mM H_2_SO_4_ as an eluent (87). Concentrations were determined from commercial standards, which were analyzed on the same run. These data were then used to calculate specific uptake and formation rates and biomass yields for propionate, succinate, and secreted by-products, respectively (Data Set S2).

### Calculation of redox cofactor and ATP balances.

Total production of reduced cofactors was determined by summing up all cofactor-forming fluxes, taking into account substrate-dependent cofactor specificities (88–90). Anabolic NADPH requirements and anabolically produced NADH were estimated from the biomass composition (75, 91) and measured specific growth rates. Surplus NADPH was considered to be converted into NADH via the activities of soluble (SthA, PA2991) and membrane-bound, proton-translocating (PntAB, PA0195-PA0196) pyridine nucleotide transhydrogenases (35).

**(i) ATP.** The total ATP demand was calculated by summing up (i) the anabolic demand needed for biomass building block synthesis and (ii) polymerization estimated from cell composition multiplied by the corresponding specific growth rate on each substrate (20, 75). We also took into account the costs of growth-associated maintenance (GAM) and nongrowth-associated maintenance (NGAM) (91) and ATP costs for substrate activation; the full reaction reference network is shown in Data Set S2 (92). The ATP synthesized by oxidative phosphorylation via the respiratory chain was estimated assuming a P/O ratio of 1.875 for NADH and PQQH_2_ (93) and 1.0 for FADH_2_ and other quinone (QH_2_) carriers (94), respectively. The anabolic ATP requirement was calculated from published biomass composition data for pseudomonads (mainly protein, RNA, and lipid synthesis) inclusive of the costs of polymerizing the precursors of these components (75, 95). The GAM and NGAM costs for pseudomonads were previously modeled using genome-scale models (91, 95). Here, an ATP surplus represents the amount of ATP available to fulfil remaining cellular ATP-consuming tasks.

### Western blotting analysis.

Equal amounts of protein (10 μg) were resolved on a 12% SDS-PAGE gel. The resolved proteins were blotted onto a nitrocellulose membrane, which was blocked with 5% (wt/vol) skimmed milk powder in Tris-buffered saline (TBS) buffer. The membranes were probed with rabbit-derived anti-ICD antibodies, anti-PrpC antibodies, or anti-PrpB antibodies (polyclonal antibodies raised by BioGenes). Following washing to remove excess primary antibody, the membranes were then probed with IRDye 800CW goat anti-rabbit IgG secondary antibodies (926-32211). Bands were visualized on an Odyssey Infrared Imaging System (LI-COR Biosciences).

### Enzymatic assays.

The 2-methylcitrate synthase (2-MCS) activity of PrpC was measured using a method described by Srere et al. (96) except that propionyl-CoA (PrCoA) was used instead of acetyl-CoA (AcCoA). Briefly, the condensation reaction of oxaloacetate (OAA) and PrCoA facilitated by PrpC generates free coenzyme A (CoA). The free CoA thiol group on the liberated CoA reacts with 5,5′-dithiobis(2-nitrobenzoic acid) (DTNB) to yield 2-nitro-5-thiobenzoate (TNB^2–^) anions. TNB^2–^ is colored, and its formation can be monitored at 412 nm. The initial rate was calculated from the rate of change of the absorbance at 412 nm (A_412_) assuming an extinction coefficient for TNB^2–^ of 14,150 M^−1 ^cm^−1^. The reaction mixtures contained buffer (50 mM HEPES pH 7.5, 0.1 M KCl, and 0.54 M glycerol), substrates (OAA and PrCoA at the indicated concentrations), and 0.15 mM DTNB. Reaction mixtures were equilibrated at 37°C for 5 min before the reaction was initiated by the addition of PrpC (to a final concentration of 240 nM). The reaction mixture was kept at 37°C, and the A_412_ was measured in a BioSpectrometer (Eppendorf). Kinetic parameters were calculated using best-fit nonlinear regression and plotted using GraphPad Prism version 6. The citrate synthase (CS) activity of PrpC was measured using the method above but with AcCoA in place of PrCoA. The 2-MCS and CS activity of GltA was measured using PrCoA and AcCoA, respectively.

### Protein expression.

The PCR-amplified ORFs of *prpC*, *gltA*, and *prpR* were cloned into the expression vector pET-19m, which introduces a tobacco etch virus (TEV)-cleavable N-terminal hexahistidine tag onto each protein. For purification of the His_6_-tagged proteins, the cells were grown in LB medium (1 L) at 37°C with good aeration to absorbance at 600 nm (*A*_600_) of 0.5. The temperature was then lowered to 16°C, and isopropyl 1-thio-β-d-galactopyranoside was added to 1 mM final concentration to induce expression of the cloned genes. The induced cultures were grown for a further 16 h and then harvested by sedimentation (6,000 × *g*, 4°C, 15 min). The cell pellet was resuspended in 20 mL of buffer A (50 mM sodium phosphate, 100 mM NaCl, and 10% (vol/vol) glycerol [pH 8.0] containing one dissolved cOmplete EDTA-free protease inhibitor cocktail tablet [Roche]), and the cells were ruptured by sonication (3 × 10 s, Soniprep 150, maximum power output). The cell lysate was clarified by centrifugation (11,000 × *g*, 4°C, 30 min), and the supernatant was filtered through a 0.45-μm filter. The filtered lysate was then loaded onto a 5-mL nickel-nitrilotriacetic acid (Ni-NTA) Superflow column (Qiagen), and the column was washed with buffer A containing 10 mM imidazole. The His_6_-tagged proteins were eluted with buffer A containing 250 mM imidazole. His_6_-tagged TEV protease (1 mg) was added to the purified protein solution, and the mixture was dialyzed overnight at 4°C against 2 L of buffer B (20 mM Tris-HCl, 50 mM NaCl, 5% [vol/vol] glycerol, pH 7.5). Uncleaved protein and the His_6_-TEV protease were removed by batch extraction in a slurry of Ni-NTA resin equilibrated in buffer B. The unbound (cleaved) protein was concentrated to the desired concentration using an Amicon Ultra-4 centrifugal filter (10-kDa molecular weight cutoff).

### Protein crystallization.

**(i) PrpC.** Crystallization conditions were screened using the sitting drop vapor diffusion technique with approximately 13 to 15 mg/mL purified PrpC solution. Optimization conditions were prepared using the dragonfly discovery system (TTP LabTech). Protein drops were generated using an automated nanoliter liquid handler mosquito high-throughput screening (HTS) (TTP LabTech). PrpC apo and holo (OAA-bound) crystals were obtained in a 1:1 ratio of protein and reservoir solution (100 to 200 mM Bis-Tris [pH 5.5], 20 to 30% [wt/vol] PEG 3350, and 0.1% d-xylose). To obtain the OAA-bound structure of PrpC, the crystallization solution additionally contained 2.5 mM oxaloacetate. All crystals were grown for 2 to 11 days at 19°C and were cryoprotected with 25% (vol/vol) glycerol and 75% (vol/vol) reservoir solution before mounting in nylon loops (Hampton Research). Mounted crystals were flash-frozen in liquid nitrogen before data collection.

**(ii) GltA.** Purified GltA at a concentration of 20 to 25 mg/mL was crystalized by sitting drop vapor diffusion. Crystals were grown for 7 days at 19°C and were cryoprotected with 25% (vol/vol) glycerol before being mounted and flash-frozen for data collection.

### X-ray diffraction, structure determination, and refinement.

**(i) PrpC.** Diffraction data were collected on beamline MX-I03 at the Diamond Light Source Synchrotron (DLS; Didcot, UK). The parameters for the data collection were as follows: omega (Ω) start: 62.0°; Ω oscillation: 0.10°; total oscillation: 180°; total images: 1,800; and exposure time: 0.050 s. Diffraction images were processed using Xia2 DIALS (97). The structure was determined by molecular replacement using Phaser (98) with the atomic coordinates of the PrpC from Coxiella burnetii (PDB entry 3TQG) as the search model. Automated refinement was performed using Refmac5 (99) and PHENIX.refine (100). Manual modeling and refinement were performed in COOT (101). Data collection and refinement statistics are listed in Table S1D.

**(ii) GltA.** Data were collected on MX-I03 beamline at the Diamond Light Source synchrotron (DLS, Didcot, UK). The parameters for the data collection were as follows: omega (Ω) start: 0°; Ω oscillation: 0.20°; total oscillation: 240°; total images: 1,200; and exposure time: 0.050s. Diffraction images were processed using Xia2 DIALS (97). The structure was determined by molecular replacement using Phaser (98) with the atomic coordinates of the type II citrate synthase from Vibrio vulnificus (PDB entry 4E6Y) as the search model. Automated refinement was performed using Refmac5 (99) and PHENIX.refine (100). Manual modeling and refinement were performed in COOT (101). Data collection and refinement statistics are listed in Table S1D.

### Analytical ultracentrifugation.

Analytical ultracentrifugation-sedimentation velocity (AUC-SV) was conducted in the Department of Biochemistry (University of Cambridge) Biophysics Facility. Samples were dialyzed overnight at 4°C against a buffer solution containing 100 mM NaCl and 50 mM Tris-HCl (pH 7.5) to remove traces of glycerol. Data were collected using an An60Ti analytical rotor (Beckman Coulter) in a Beckman Optima XL-I ultracentrifuge with absorbance and interference optical detection systems. Protein solution (40-μL volume and concentration of approximately 1 mg mL^−1^) and the reference solution (dialysate) were added to the Epon (epoxy) double-sector centerpieces. All samples were sedimented at 40,000 rpm and 20°C. Absorbance data (*A*_280_) were collected in intervals of 2 min, and interference scans were taken every 1 min. The viscosity and density of the buffer used in the experiments were estimated with SEDNTERP. Data analysis was conducted using SEDFIT.

### Protein thermal stability.

Differential scanning fluorimetry experiments were carried out using a CFX Connect RT-PCR detection system (Bio-Rad) and using Hard-Shell 96-well PCR plates (Bio-Rad), which are compatible with the excitation and emission wavelength of SYPRO orange. The temperature range was 4 to 95°C with an increment of 1°C every 45 s. The fluorescence was measured every 15 s. Data were analyzed using GraphPad Prism 6.

### EMSA analysis.

The region upstream of *prpR* (250 bp, including the 12-nucleotide palindrome) was PCR amplified. The forward primer contained a 6-carboxyfluorescein (6-FAM) tag (Sigma). EMSA reaction mixtures (25-μL volume) contained buffer (20% [vol/vol] glycerol, 30 mM Tris-HCl [pH 8.0], 1 mM MnCl_2_, 120 mM KCl, and 1 mM MgCl_2_) supplemented with 5 pM 6-FAM-labeled probe, up to 2 μM recombinant His_6_-PrpR, 240 μg/mL bovine serum albumin (BSA), and 15.2 μg/mL Poly-deoxy-inosinic-deoxy-cytidylic acid (poly(d[I-C])). After incubation at 21°C for 60 min, individual samples were applied to a 6% polyacrylamide gel (Novex) prepared in Tris-borate-EDTA buffer. The samples were electrophoresed in the same buffer system for 45 min at 120 V. The gels were then imaged using an Odyssey imager (Li-Cor Biosciences). In the competition EMSA, unlabeled competitor probes harboring specific nucleotide substitutions were added in 50-fold molar excess relative to the labeled probes.

### LC-MS analysis of propionyl-CoA.

Sampling, analysis, and quantification of propionyl-CoA and other CoA esters was carried out as described previously (102). Briefly, cells from 8-mL cultures grown to an OD_600_ of 2 were pelleted and resuspended in 200 μL of “supercool” ultrapure water (0°C) and 1 mL of quenching-extraction buffer (95% acetonitrile and 25 mM formic acid at −20°C). The mixture was vortexed then kept on ice for 10 min and finally centrifuged (3 min at 0°C). The supernatants were transferred into 3 mL of ultrapure water, then and snap-frozen in liquid nitrogen and lyophilized (Alpha 3-4 LSCbasic, Christ, Germany). The freeze-dried samples were diluted in 500 μL of precooled resuspension buffer (25 mM ammonium formate [pH 3.0] and 2% methanol at 4°C) and immediately analyzed by LC-MS (a QTRAP 6500+ [AB Sciex, Darmstadt, Germany] coupled to a high-performance liquid chromatography (HPLC) system (Agilent Infinity 1290)). Commercial standards were used for quantification. Final concentrations are given as nmol per gram dry cell weight (DCW).

### Data availability.

The sequencing data are deposited at ArrayExpress (accession number E-MTAB-10077).
