# Supplementary figures and images for: Systems-Wide Dissection of Organic Acid Assimilation in Pseudomonas aeruginosa Reveals a Novel Path To Underground Metabolism
Source: mBio. 2022 Nov 15;13(6):e02541-22. doi: 10.1128/mbio.02541-22 (PMC9765439; doi:10.1128/mbio.02541-22)

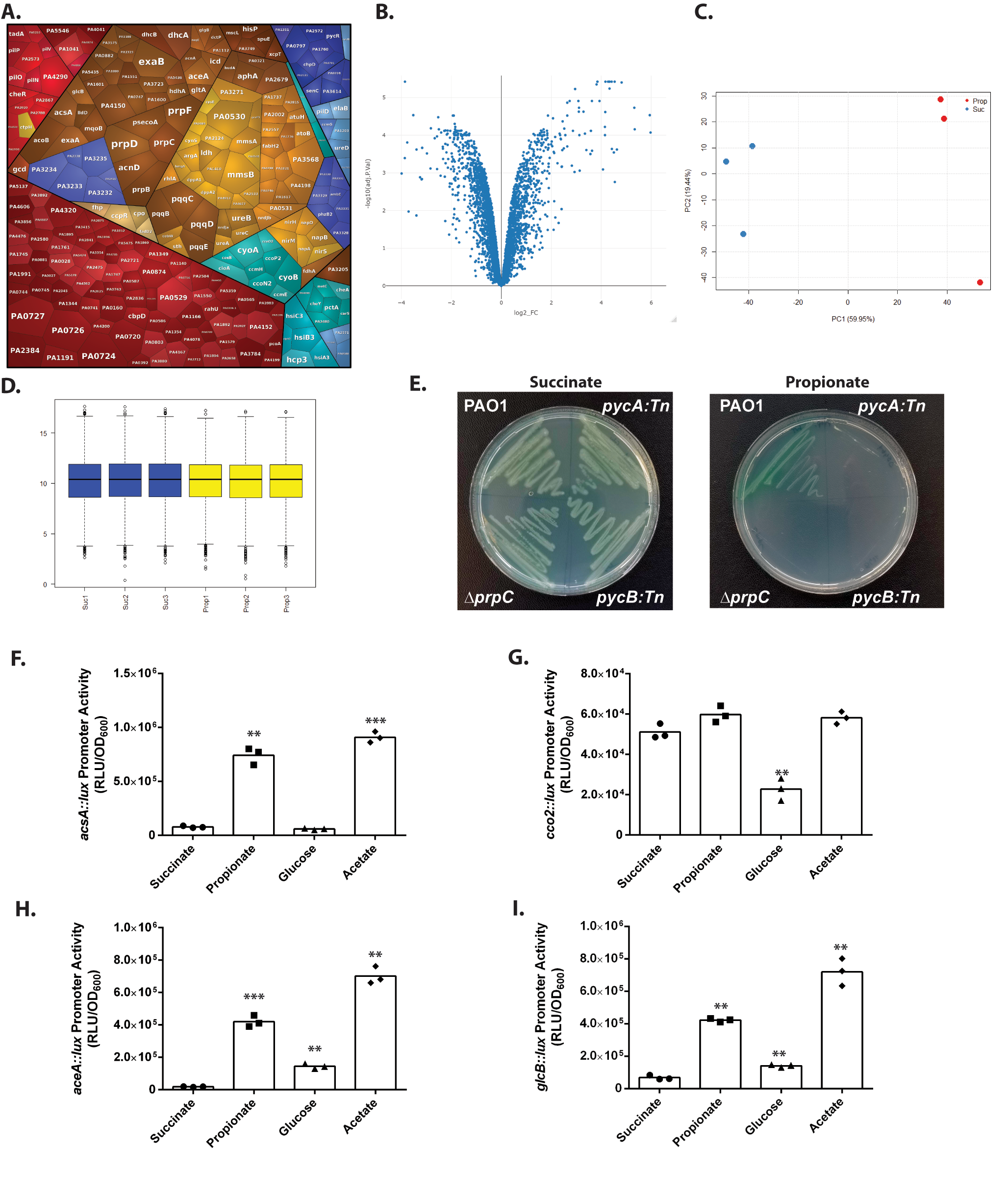

Supplement: FIG S1 [file mbio.02541-22-sf001.png]

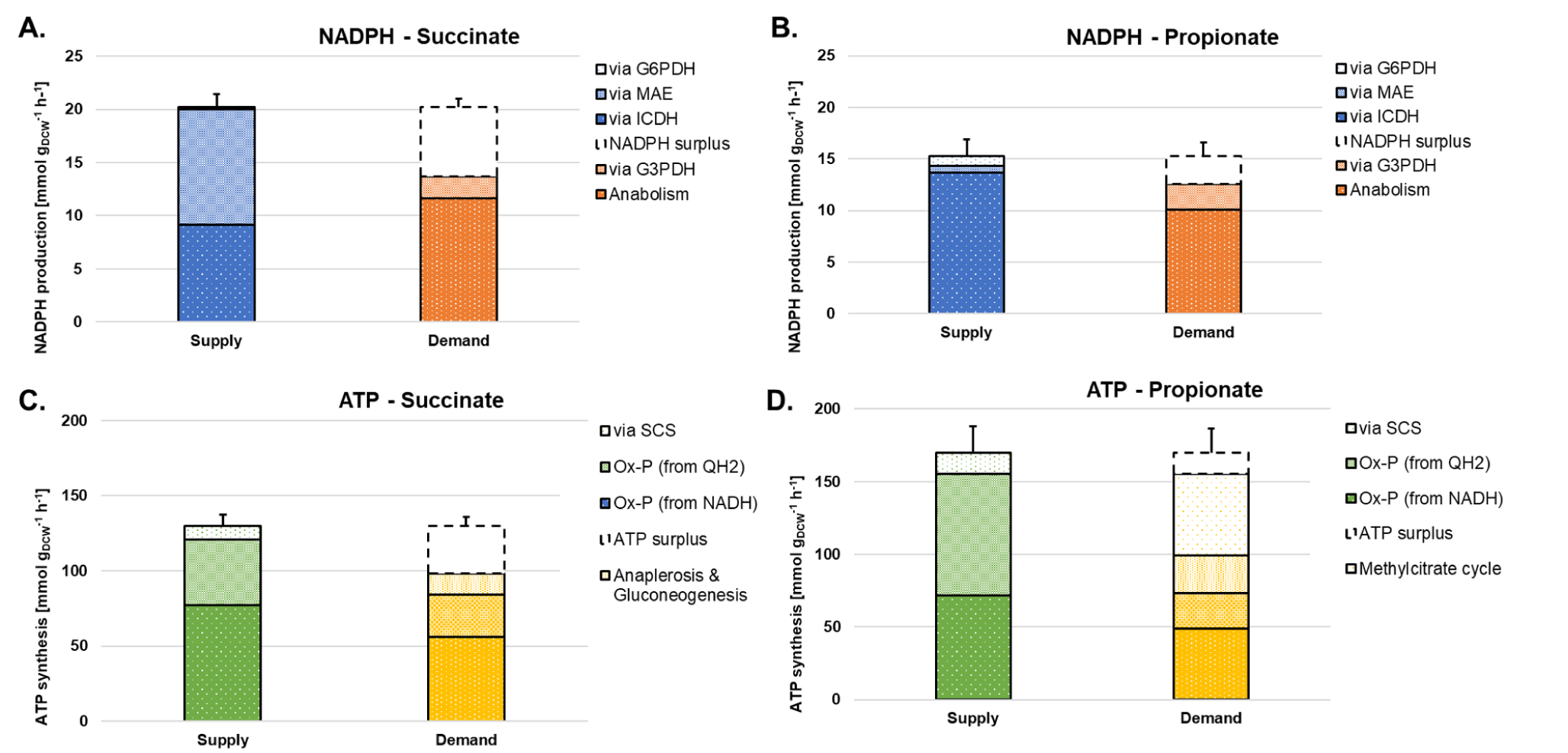

Supplement: FIG S2 [file mbio.02541-22-sf002.png]

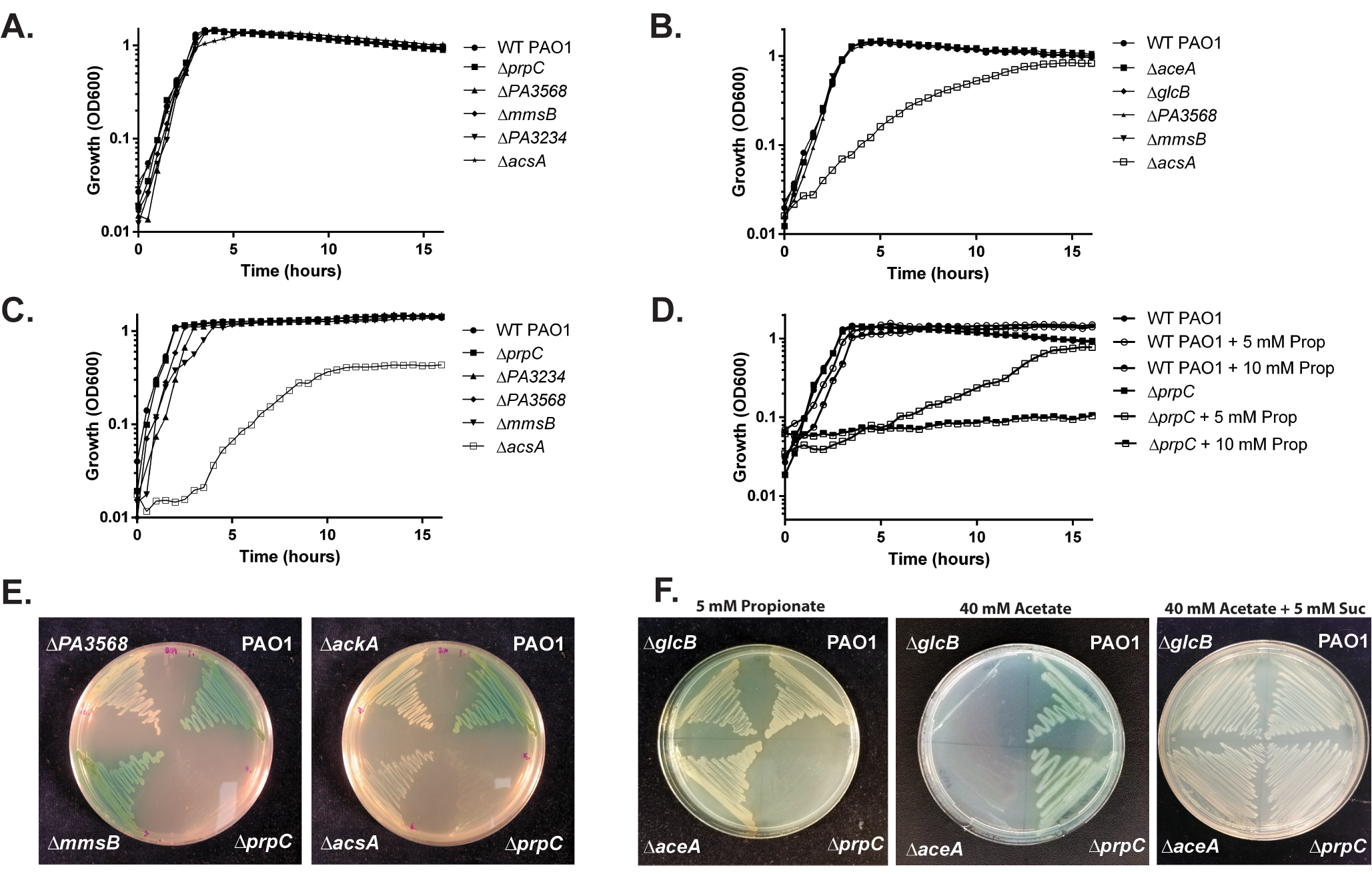

Supplement: FIG S3 [file mbio.02541-22-sf003.png]

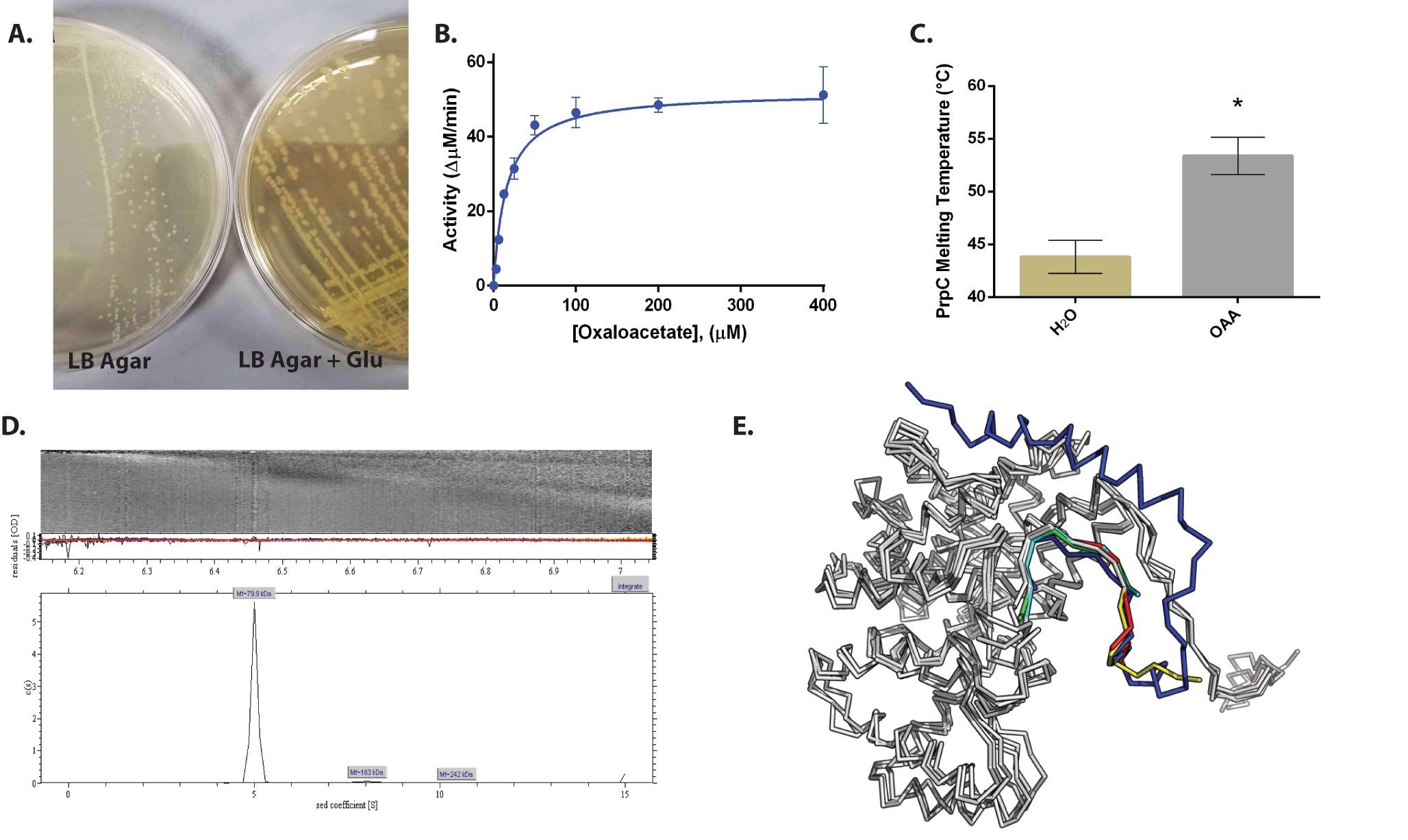

Supplement: FIG S4 [file mbio.02541-22-sf004.tif]

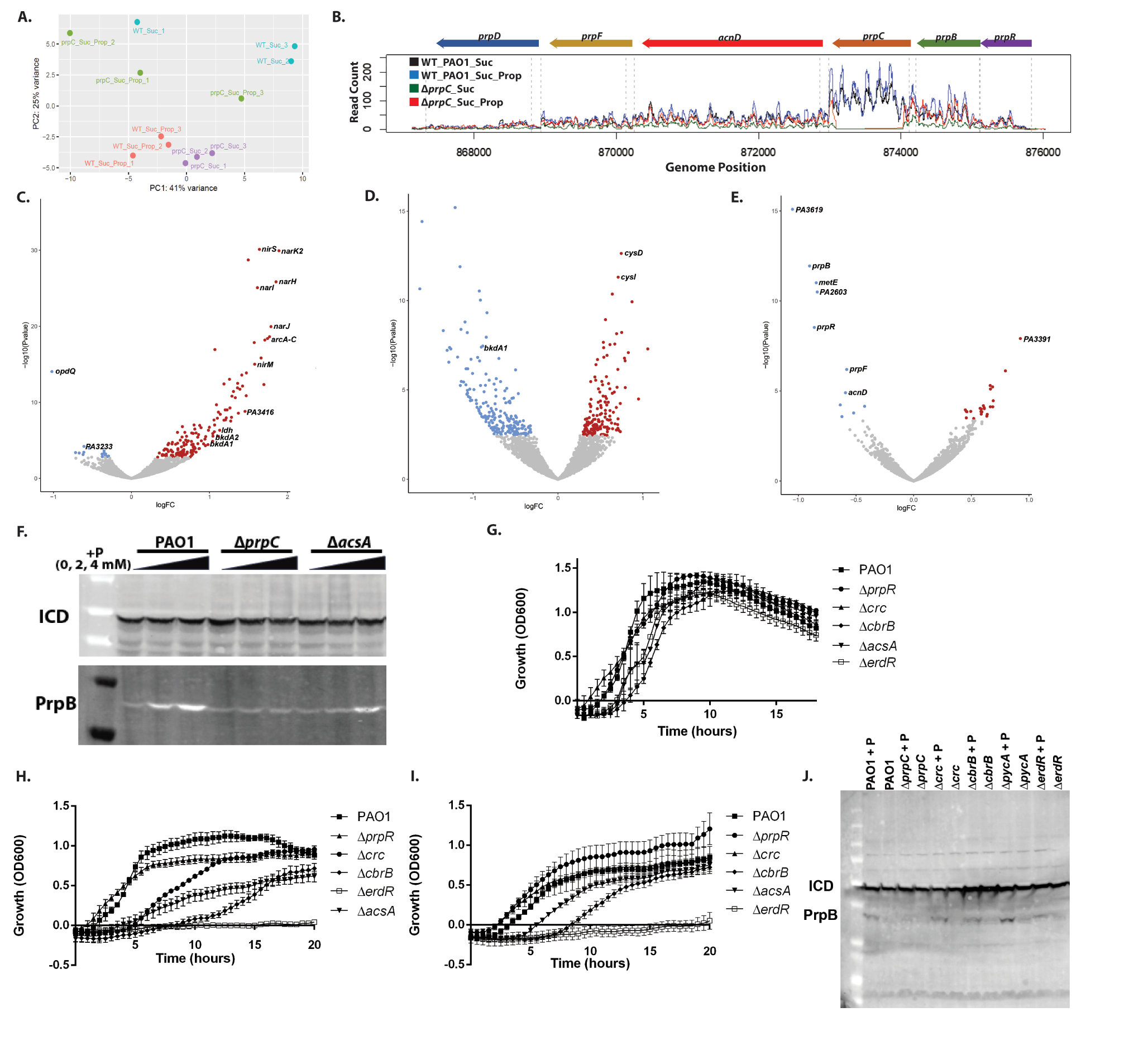

Supplement: FIG S5 [file mbio.02541-22-sf005.png]

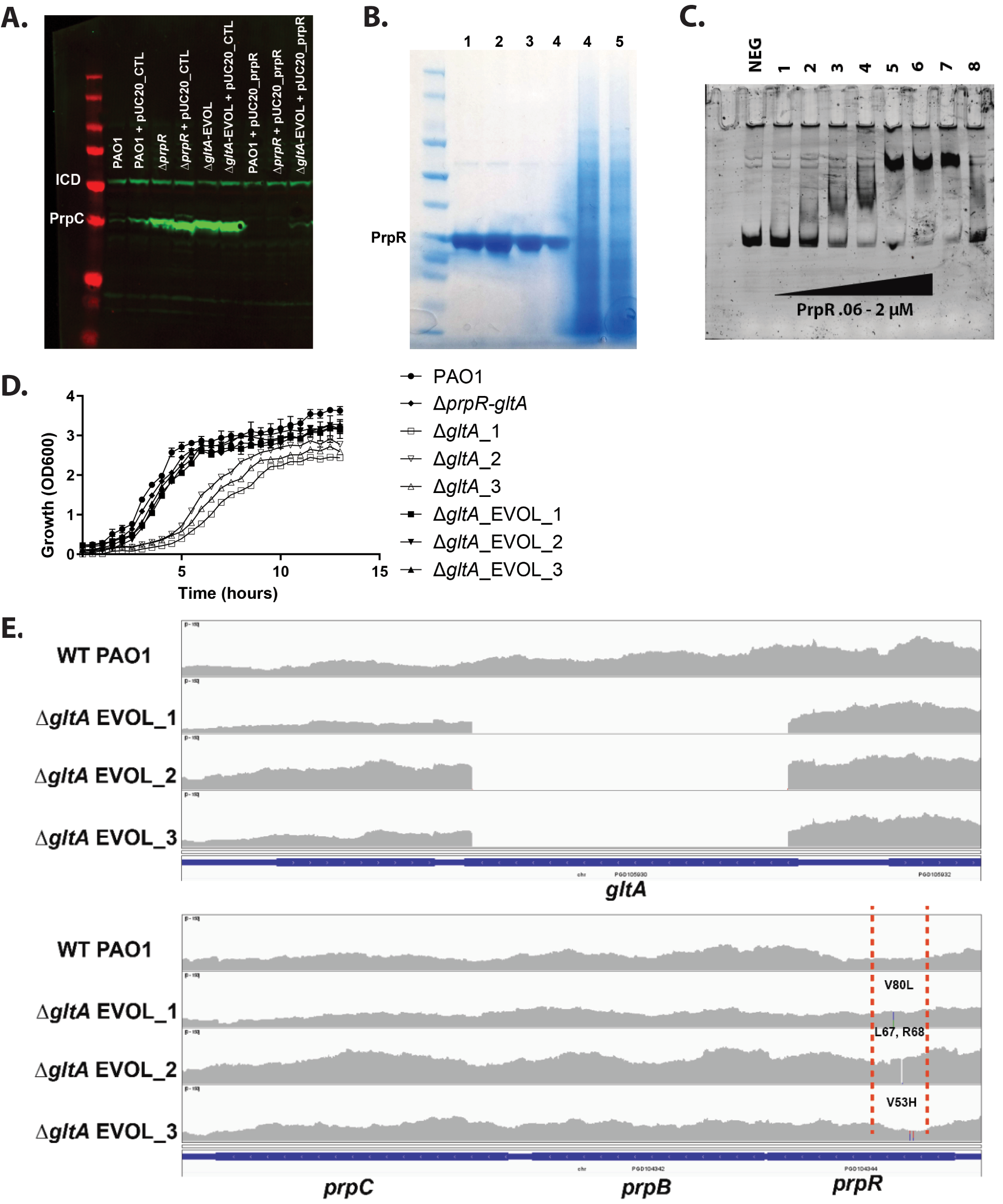

Supplement: FIG S6 [file mbio.02541-22-sf006.png]
